# PROTOCOL: Key characteristics of effective preschool‐based interventions to promote self‐regulation: A systematic review and meta‐analysis

**DOI:** 10.1002/cl2.1383

**Published:** 2024-04-02

**Authors:** Atsushi Kanayama, Iram Siraj, Mariola Moeyaert, Kat Steiner, Elie ChingYen Yu, Katharina Ereky‐Stevens, Kaoru Iwasa, Moeko Ishikawa, Mehar Kahlon, Rahel Warnatsch, Andreea Dascalu, Ruoying He, Pinal P. Mehta, Natasha Robinson, Yining Shi

**Affiliations:** ^1^ Department of Education University of Oxford Oxford UK; ^2^ Department of Educational and Counseling Psychology The State University of New York Albany New York USA; ^3^ Bodleian Health Care Libraries University of Oxford Oxford UK; ^4^ Division of Educational Psychology and Methodology The State University of New York Albany New York USA; ^5^ Independent Researcher Ikeda Osaka Japan; ^6^ Graduate School of Human Sciences Osaka University Osaka Japan; ^7^ FirstSteps IB World School Chandigarh India; ^8^ Independent Researcher Cologne Germany; ^9^ Independent Researcher Oxford UK; ^10^ Division of the Social Sciences University of Chicago Chicago Illinois USA; ^11^ School of Education University of Bristol Bristol UK; ^12^ Department of Psychology University of Cambridge Cambridge UK

## Abstract

This is the protocol for a Cochrane Review. The objectives are as follows: The aim of this systematic review is to advance our understanding of the key characteristics of effective preschool‐based interventions designed to foster self‐regulation. To accomplish this, the review addresses the following questions: 1. What types of preschool‐based interventions have been developed to promote self‐regulation? 2. What is the average effect of these preschool‐based interventions on self‐regulation, focusing on four key constructs: integrative effortful control, integrative executive function, self‐regulation, and self‐regulated learning? 3. What characteristics—such as Resource Allocation, Activity Type, and Instruction Method—could potentially contribute to the effects of preschool‐based interventions in promoting self‐regulation?

## 1 BACKGROUND

### 1.1 Description of the condition

We stand at the crossroads of a transformative era, where digital evolution extends beyond mere technology, deeply intertwining with nuances of social dynamics. Emergent digital networks redefine how we live, work, and relate to each other, dissolving traditional boundaries and placing us within a global web of interconnectivity (Castells, 2010; David & Foray, 2002; van Dijk, 2020). This rapid social change calls for adaptability, while also spotlighting the need for richer social interactions and collaborative endeavors, particularly among individuals with diverse ethical perspectives and values (Benner, 2004; Martindale & Lehdonvirta, 2021).

As technology becomes increasingly integral to our everyday lives, the urgency for universal access to digital resources becomes apparent, as does the necessity for a robust suite of competencies that extend beyond the digital realm. Traversing this expansive digital landscape requires the prowess to critically evaluate, create, and communicate information, as well as a commitment to lifelong learning (Grafstein, 2002; Hurd, 1998; Leaton Gray, 2017; Leaton Gray et al., 2022). Complicating matters, the “learning divide,” a byproduct of socio‐economic and educational disparities, introduces multifaceted challenges (Gorard et al., 2003; P. White, 2012). Overcoming this divide necessitates the cultivation of 21st‐century skills, including digital literacy, critical thinking, problem‐solving, adaptability, and resilience (Loble et al., 2017). At the heart of this skillset is the need to nurture autonomy and creativity in learners, thereby empowering them to not only adapt but also pioneer future trajectories (Deci et al., 2017; R. M. Ryan & Deci, 2006).

Self‐regulation denotes the multi‐dimensional, self‐directed ability to align one's thoughts, emotions, and behaviors in response to both internal factors—such as motivations, emotional states, and physiological cues ‐ and external factors—such as social and environmental conditions. This ability enables individuals to adeptly navigate changing circumstances across time and space, harmonize immediate needs and desires with overarching goals, and adjust beliefs, values, and strategies in light of new insights according to individual will (Callan, 2018; Eccles & Wigfield, 2002; Nigg, 2017; R. M. Ryan & Deci, 2006; Zimmerman, 2000). Gaining recognition from academia, educators, policymakers, and business leaders, self‐regulation emerges as a foundational pillar of contemporary competencies, steering us through the myriad challenges of our dynamic landscape (R. E. Anderson, 2008; Geisinger, 2016; Loble et al., 2017). The COVID‐19 crisis magnified its pivotal role, as children faced unparalleled socio‐emotional and behavioral challenges, such as diminished playful interactions, feelings of isolation, and abrupt changes to their daily routines. These adversities underscored the indispensable nature of self‐regulation in steering through uncertainty and adapting to evolving social norms.

Anchored in the cybernetic model, self‐regulation is built upon a hierarchy of goals that span from broad abstract ideals down to concrete, actionable objectives (Carver & Scheier, 1998, 2000a, 2000b, 2012, 2016). In line with the above definition of self‐regulation, this model underscores the significance of harnessing information from our internal states and external environment to steer our behaviors. Our present states are juxtaposed against established benchmarks embedded within our internal goal structure. This ongoing self‐assessment refines our goals, synchronizes our current states with them, and monitors our progress. Feedback mechanisms play a pivotal role in adjusting strategies to align with evolving goals. Emotions serve as vital cues, prompting a reevaluation of goals and reallocating resources when needed. The cybernetic model, by emphasizing the dynamic interplay between spontaneous reactions (bottom‐up control, including gating mechanisms) and deliberate, goal‐oriented actions (top‐down control; Carver & Scheier, 2012, 2016; Nigg, 2017), offers a multifaceted and nuanced lens through which to view self‐regulation.

This review examines four key constructs closely linked to the emergence of top‐down control, a cornerstone of self‐regulation that becomes evident in the preschool years. It bridges basic cognitive processes such as effortful control and executive functions, key areas of focus in developmental psychology (Allan & Lonigan, 2011; Diamond, 2013; Garon et al., 2008; Ishikawa et al., 2023; Kim et al., 2013; Kochanska & Knaack, 2003; Nigg, 2017; Rothbart & Bates, 2006; H. Schmidt et al., 2022; Tiego et al., 2020; Zhou et al., 2012), with advanced strategies that encompass complex cognitive processes, such as self‐regulation and self‐regulated learning, which have garnered considerable attention in educational research (Dinsmore et al., 2008; Efklides, 2011; Flavell, 1979; Livingston, 2003; Post et al., 2006; Whitebread et al., 2007; Whitebread, Coltman, Pasternak, et al., 2009; Zimmerman & Schunk, 1989). This synthesis provides a nuanced perspective on the current state of evidence concerning the readily observable facets of self‐regulation, specifically focusing on how children develop their abilities to regulate thoughts, emotions, and behaviors, and employ strategies to achieve their goals.

Effortful control is a concept deeply rooted in temperament research and is recognized as one of the earliest self‐regulatory abilities developed in childhood (Kälin & Roebers, 2021; Nigg, 2017; H. Schmidt et al., 2022). This construct involves the skillful use of executive attention and encompasses an individual's ability to suppress a dominant response, promote a subdominant response, formulate plans, and recognize errors (Rothbart, 2012; Rothbart & Bates, 2006). While the primary context of effortful control is intertwined with emotion regulation, its influence also extends to non‐emotional tasks such as delaying tasks (e.g., Snack Delay), motor inhibition tasks (e.g., Walk‐a‐Line Slowly), suppressing‐initiating response to signal tasks (e.g., Go/No Go task), and effortful attention tasks (e.g., Stroop‐ like task; Allan & Lonigan, 2011; Kim et al., 2013; Kochanska & Knaack, 2003; Zhou et al., 2012).

Executive function, a central focus of neurocognitive research, refers to higher‐order cognitive operations that direct our thoughts, emotions, and actions toward achieving goals, particularly in non‐routine situations (Banich, 2009; Diamond, 2013; Garon et al., 2008; R. Jacob & Parkinson, 2015; H. Schmidt et al., 2022; Traverso et al., 2015). Traditionally, executive function includes three interrelated components: working memory, inhibition, and shifting (Hofmann et al., 2012; McClelland, Cameron, Wanless, et al., 2007; Miyake et al., 2000; Rueda et al., 2005). However, the structural understanding of executive function remains a highly controversial topic. Some studies advocate a single‐factor model for children up to 7 years of age (Brydges et al., 2012; Shing et al., 2010; Wiebe et al., 2008; Willoughby et al., 2012), while others propose a multi‐factor model (M. D. Lerner & Lonigan, 2014; Miller et al., 2012; Schoemaker et al., 2012; Usai et al., 2014). Despite ongoing debates, executive function as a component of fluid cognitive abilities may exhibit developmental adaptability, whereas general intelligence tends to be stable across the lifespan (Blair, 2006; Blair & Raver, 2015; Garlick & Sejnowski, 2006; Heitz et al., 2006).

Effortful control and executive function exhibit significant overlap, particularly in the context of self‐regulation among preschoolers (Garon et al., 2008; McKenna et al., 2017; H. Schmidt et al., 2022). McKenna et al. (2017) put forth a developmental model that highlights the partially distinct yet interconnected components of executive function. Contrasting this, Howard et al. (2015) argue for the potential integration of these functions during the preschool years. Importantly, these cognitive processes are not isolated phenomena; they often involve a synergistic interplay of top‐down and bottom‐up control mechanisms, particularly in real‐world situations. As such, experts recommend comprehensive assessment tools, such as the NIH Toolbox and the Head‐Toes‐Knees‐Shoulders task for a nuanced evaluation (McClelland, Cameron, Wanless, et al., 2007; McClelland et al., 2018; McClelland & Cameron, 2011). They also advocate for targeted interventions that foster this balanced approach to cognitive control (Blair & Raver, 2015; Diamond, 2013). Embracing such an integrative methodology is crucial for effectively nurturing and assessing these cognitive skills, especially in educational environments where problem‐solving is a central focus (Howard et al., 2015; Zhou et al., 2012).

Another research stream of self‐regulation emphasizes cognitive strategies pertinent to real‐world scenarios (Nigg, 2017). Self‐regulation and self‐regulated learning, often deemed “complex” forms of self‐regulation, encompass not only the basic cognitive processes associated with effortful control and executive function but also additional cognitive and metacognitive strategies. These strategies extend beyond the realm of basic cognitive processes and involve the capability to plan, monitor, and adapt behavior in the face of changing social circumstances. Initially, the concept of self‐regulation was mainly tied to behavioral control (Bandura, 1977). However, its scope has broadened to include not just cognitive and emotional regulation but also academic learning (Post et al., 2006; Zimmerman & Schunk, 1989), a development substantiated by an extensive review of 255 studies (Dinsmore et al., 2008). More recent research has further nuanced this field by introducing social regulation as a distinct yet closely related facet of self‐regulation, especially in the context of collaborative learning environments (Grau & Whitebread, 2012; Whitebread, Coltman, Pasternak, et al., 2009). Metacognition, another critical aspect of self‐regulation, revolves around an individual's active management of cognitive processes and is rooted in Flavell's work (Flavell 1979, 1985; Livingston, 2003). Moreover, metacognition is increasingly linked with self‐regulation (Efklides, 2011; Whitebread, Coltman, Jameson, et al., 2009; Whitebread et al., 2007), making it essential to the process of monitoring and controlling cognition within the broader framework of self‐regulation or self‐regulated learning (Dinsmore et al., 2008).

The development of self‐regulation in early childhood has far‐reaching consequences that extend beyond the formative years. These abilities are pivotal for a child's overall health, socio‐emotional well‐being, academic achievement, and social competence. According to extensive research, mastering self‐regulation lays the groundwork for both immediate and long‐term positive outcomes in various aspects of life (Blair & Raver, 2015; Korucu et al., 2017; Lenes et al., 2020; McClelland et al., 2018; Robson et al., 2020; Whitebread, Coltman, Jameson, et al., 2009). Children with strong self‐regulatory abilities are better equipped to manage impulses, concentrate effectively, follow rules, overcome challenges, and maintain positive relationships with peers and teachers (Blair & Raver, 2015; Eisenberg et al., 2010; Hammer, 2018; McClelland & Cameron, 2012; Raver et al., 2011). These abilities also foster resilience, equipping children to better cope with a range of challenges, from cognitive and emotional hurdles to social complexities (Boekaerts, 1999; Crespo et al., 2019; Dias & Cadime, 2017; Gardner et al., 2008; Masuda, 1981, p. 1; Sektnan et al., 2010). The benefits are manifold: children with robust self‐regulation show not only better school readiness and remarkable academic progress (McClelland, Cameron, Wanless, et al., 2007; Raver et al., 2011; Wanless et al., 2011) but also enjoy better physical health (Francis & Susman, 2009; Moffitt et al., 2011) and are less likely to engage in criminal behavior or substance abuse in later life (Moffitt et al., 2011). It is worth noting that some children may lack these abilities, underscoring the vital importance of supportive role models and caregivers in nurturing their development (Blair et al., 2002; Grolnick, 2009; Grolnick et al., 1999; Lonigan et al., 2022; Pandey et al., 2018).

While self‐regulation development—from birth to age six—is shaped by a complicated web of environmental, sociocultural, and individual factors (see Supporting Information: Appendix 1 for a detailed overview), it is essential to understand that these abilities are not merely acquired passively. They can be actively cultivated through carefully designed interventions (Blair & Raver, 2015; Boekaerts, 1999; Schunk & Zimmerman, 2003). Contemporary research is increasingly focused on creating and assessing programs aimed at fostering self‐regulation in young children, particularly within structured educational settings. These initiatives strive not only to facilitate a smooth transition to formal schooling but also to endow children with essential life competencies that contribute to their long‐term well‐being and success (Centers for Disease Control and Prevention, 2010; R. J. Duncan et al., 2018; McClelland et al., 2015; National Association for the Education of Young Children, 2021; Schmitt et al., 2015).

### 1.2 Description of the intervention

This review explores tier‐one interventions specifically tailored for preschool settings, aiming to enhance self‐regulation among preschoolers. Designed for ease of implementation, these interventions can be effectively executed by school staff or external facilitators with minimal specialized training, making them highly adaptable across diverse preschool contexts.

The interventions encompass an array of activities designed to foster basic self‐regulation integrating key aspects of effortful control and executive function as well as more complex self‐regulatory processes including self‐regulation and self‐regulated learning. While the primary focus is on strengthening child self‐regulation, these interventions may also offer additional benefits. They systematically integrate elements targeting four core constructs of self‐regulation and employ relevant assessment measures to monitor progress.

The interventions offer significant flexibility in Resource Allocation, accommodating various factors such as participant needs, research objectives, and practical constraints. As for dosage, the intervention period can span from a few weeks to several months. Additionally, the total training volume can be adjusted based on the duration and frequency of individual sessions. Our review primarily addresses these dosage components but also recognizes the potential impact of adherence to intervention protocols—commonly known as implementation dosage—on intervention effects (Laurent et al., 2019; McCoy, 2017; Meza et al., 2020; Wasik et al., 2013). Group size factors such as class size, the number of adult facilitators, and the pupil‐teacher ratio are also modifiable, ensuring a tailored experience that meets the unique needs of each participant.

The activities employed in these interventions are grounded in self‐regulation theories and feature a diverse set of exercises, including physical movement, music, art, storytelling, pretend play, construction activities, mindfulness exercises, and academic tasks. Each exercise is carefully designed to align with children's developmental stages.

The instructional methods used in most of these interventions combine direct instruction – where teachers explicitly explain and model self‐regulation strategies – with a constructivist approach that encourages children to discover self‐regulation strategies through problem‐solving and peer collaboration (Hattie, 2009; Reynolds & Miller, 2003; Schunk & Zimmerman, 2003). As children advance in their abilities, the level of instructional support is gradually reduced, and task difficulty is adjusted to match their growing capabilities. Some interventions may also strategically use feedback and rewards to encourage active engagement and reinforce positive behavior (Hadwin, 2008; Schunk, 1983, 1984).

While these interventions primarily target individual self‐regulation, they do not aim to indirectly modify children's broader social environments or enhance the quality of teacher‐child interactions outside the training context. Unlike standard off‐the‐shelf programs, these interventions may intentionally blend various activities with the primary focus on promoting child self‐regulation. Their design enables seamless integration into regular classroom routines, offering children continuous opportunities to practice and refine their self‐regulatory abilities in their everyday learning environments.

### 1.3 How the intervention might work

This review aims to explore the complex dynamics that influence the effects of preschool‐based interventions in enhancing self‐regulation among children. We have identified three cornerstone categories—Resource Allocation, Activity Type, and Instruction Method—as the analytical lenses through which we examine the impact of various intervention characteristics on child self‐regulation, our primary outcome of interest.

Our overarching goal is to synthesize existing evidence to understand whether and how preschool‐based interventions are associated with improvements in self‐regulation. We aim to go beyond merely identifying correlations by examining the variability in outcomes. By incorporating these intervention characteristics as moderators in our meta‐regression analyses, we seek to shed light on the underlying mechanisms that may account for this variability.

It is important to clarify that this meta‐analysis is not designed to provide direct empirical evidence establishing causal links between self‐regulation (our primary outcome of interest) and academic skills (our secondary outcome). Instead, we aim to synthesize the existing literature to make informed inferences about these potential associations.

Our Theory of Change will outline the hypothesized pathways linking interventions to both primary and secondary outcomes. It is crucial to note that our exploration aims to illuminate potential mechanisms that may influence variations in self‐regulation outcomes, rather than to definitively establish causality.

#### 1.3.1 Resource allocation

##### Dosage

Dosage, traditionally understood as the planned amount of training administered during an intervention, plays a crucial role in understanding how interventions can be optimally delivered, resourced, replicated, and scaled up (Rowbotham et al., 2019; Wasik et al., 2013). Dosage also captures the notion of “the change to amount dispensed over time,” without necessarily implying linear causal assumptions (Rowbotham et al., 2019, p. 1).

Wasik et al. (2013) distinguish between two forms of dosage: intervention dosage and implementation dosage. Intervention dosage refers to the planned volume of training intended for the target group, as specified in the study design. In contrast, implementation dosage accounts for the actual volume of training delivered and received, influenced by factors such as adherence to intervention protocols (Musci et al., 2019). Implementation dosage has been shown to predict outcomes such as teacher adherence (Meza et al., 2020) and student engagement (Laurent et al., 2019).

Our review examines how intervention effects may vary based on both types of dosage. When we use the term “dosage,” we are specifically referring to “intervention dosage,” in accordance with the intention‐to‐treat principle (McCoy, 2017). Additionally, we intend to examine the influence of implementation levels on these effects.

By taking into account both forms of dosage, we strive for a nuanced understanding of the intervention's effectiveness and its applicability in real‐world settings. This dual focus enables us to interpret the outcomes of the intervention from both a design and practical implementation standpoint.

Limited yet significant evidence exists regarding the relationship between dosage and intervention outcomes. For instance, some studies indicate that higher dosages may be more effective in interventions targeting executive function (Davis et al., 2007; Diamond, 2012; Tang et al., 2012; Watson et al., 2017). Research on mindfulness‐based interventions has also found a positive relationship between training duration and the efficiency of the executive attention network in relation to self‐regulation (Tang et al., 2007, 2009). However, it is worth noting that Tang et al.'s (2012) findings were based on undergraduate students in the US, and caution should be exercised when generalizing these findings to different demographics, such as preschool children. Additionally, Davis et al. (2007) found that overweight nine‐year‐olds who participated in 40‐min exercise sessions five days a week for 15 weeks showed greater improvements in executive functions compared to their counterparts who exercised for only 20 minutes with the same frequency.

##### Group size

The impact of group size on intervention effects is a subject of ongoing debate. While smaller groups are generally favored for their potential to offer more individualized support, feedback, and opportunities for relationship‐building (Solheim & Opheim, 2019), the research findings are not universally conclusive. For instance, some studies suggest that teachers may not significantly alter their teaching practices in smaller classes, thereby casting doubt on the efficacy of reducing class size as a strategy for improved learning outcomes (Hattie, 2009). Another study indicated only a small effect of class size on reading achievement and a negligible effect on mathematics achievement (Filges et al., 2018).

However, classroom dynamics are influenced by more than just the number of students. To fully grasp the implications of class size on learning outcomes, it is necessary to look more closely at its interplay with key classroom processes such as student engagement, relationships with classmates, instructional practices, and classroom management (Blatchford & Russell, 2020).

Recognizing the potential significance of class size in the context of preschool self‐regulation interventions, we plan to explore this aspect in our meta‐analysis. Given the nuanced and context‐dependent nature of the debate surrounding classroom size, this review refrains from taking a definitive stance but emphasizes the need for additional empirical research.

While the existing literature is inconclusive, both dosage and group size could be important factors influencing the effects of preschool self‐regulation interventions. To provide a more comprehensive understanding, our review will employ meta‐regression analyses that consider the following intervention characteristics under Resource Allocation:

◆ Period: The length of the intervention in days or weeks
◆ Volume: The cumulative minutes (total duration) of training
◆ Duration: The length of individual training sessions in minutes
◆ Frequency: The number of training sessions conducted per week
◆ Adherence: The actual amount of planned training received by the children, if available
◆ Class Size: The number of children in the class during the intervention
◆ Number of Adults: The number of adults present in the classroom during the intervention
◆ Pupil‐Teacher Ratio: The ratio of students to teachers during the intervention

By employing meta‐regression analyses, we aim to examine how these Resource Allocation characteristics are associated with the effect sizes, contextualized by a critical review of existing literature. This approach will allow for a nuanced understanding of associations between Resource Allocation factors such as dosage and group size and the effects of preschool self‐regulation interventions.

#### 1.3.2 Activity type

Emerging research offers valuable insights into the potential of various activities for fostering self‐regulation among preschool children. In this section, “Activity Type” encompasses both the theoretical frameworks that inform them as well as the nature of the activities.

##### Theoretical foundations

Interventions anchored in self‐regulation theory have been shown to significantly impact their outcomes. Zimmerman's three‐phase model of self‐regulated learning—encompassing preparation, performance, and appraisal—is a prevalent framework in primary and secondary school interventions (Panadero, 2017). Meta‐analyses reveal that interventions employing social‐cognitive theory or a blend of social‐cognitive and metacognitive theories produce the most substantial effects, while those based on motivational theories demonstrate more modest effects (Dignath et al., 2008; Dignath & Büttner, 2008). Self‐Determination Theory offers another perspective on self‐regulation, conceptualizing it as goal selection in harmony with individual needs and values (Day et al., 2022). Moreover, Vygotsky's socio‐cultural perspective, which underlies the Tools of the Mind curriculum, provides valuable insights into self‐regulation (Barnett et al., 2008). Nonetheless, the effectiveness of specific interventions in real‐world settings can vary, highlighting the necessity for continued research.

##### Activity variants

Physical activities, notably those requiring a blend of working memory, inhibition, and shifting, are shown to enhance executive function and self‐regulation in young children (Becker et al., 2014; Diamond, 2012). An interesting avenue of research explores active play during outdoor preschool recess, revealing that it contributes positively to self‐regulation, emergent literacy, and math skills (Becker et al., 2014). These activities appear to enhance academic achievement, with self‐regulation playing a moderating role.

Music‐based activities provide a conducive context for self‐regulatory growth (Williams, 2018; Williams & Berthelsen, 2019; Zachariou & Whitebread, 2015, 2017, 2019). Combining music play with rhythmic body movements has been observed to indirectly foster self‐regulation through improved beat synchronization, motor coordination, relaxation, emotional regulation, and executive function (Williams, 2018; Williams & Berthelsen, 2019). Studies conducted in the UK and Cyprus lend empirical support to these benefits (Zachariou & Whitebread, 2015, 2019).

Furthermore, open‐ended activities are defined as activities without a fixed or predetermined outcome, allowing children the freedom to explore, create, and learn in a flexible environment. Examples of such activities include pretend play and construction play, which are naturally engaging for children and serve as effective platforms for developing self‐regulation (Berk et al., 2006; Berk & Meyers, 2013; Braund & Timmons, 2021; Whitebread & O'Sullivan, 2012). Compared to more structured activities with predetermined goals, open‐ended activities have been found to be particularly beneficial in fostering verbal self‐regulation. Additionally, storybooks serve as another form of open‐ended, child‐directed activity, offering opportunities for pretend play that fosters exploration, expression, and the learning of self‐regulatory strategies (Rowe, 1998; Welsch, 2008).

Mindfulness training, another Activity Type, has shown promise in strengthening self‐regulation by enhancing the mind‐body connection. The benefits can be amplified when combined with physical exercise (Diamond & Lee, 2011; Razza et al., 2015; Tang et al., 2012).

Lastly, academic activities with embedded strategy instruction can be advantageous for boosting self‐regulation in a school context. This approach aligns with the social cognitive view of learning, focusing on observation, emulation, and self‐reflection (Bandura, 1977, 1986; Schunk & Zimmerman, 2007). It also fits with Panadero's proposed framework for designing self‐regulated learning interventions, focusing on the preparation, performance, and appraisal phases (Panadero, 2017).

Overall, activities aimed at fostering self‐regulation in preschoolers come in various forms and are informed by diverse theoretical frameworks. To provide a more nuanced understanding of how these activities and frameworks influence self‐regulation outcomes, our meta‐regression analyses will investigate the following specific characteristics under Activity Type:

◆ Social Cognitive Theory‐Based Activities (e.g., Zimmerman's three‐phase model of self‐regulation)
◆ Motivational Theory‐Based Activities (e.g., Self‐Determination Theory)
◆ Socio‐Cultural Theory‐Based Activities (e.g., Vygotsky's socio‐cultural perspective)
◆ Physical Activities
◆ Musical Activities
◆ Pretend Play Activities
◆ Construction Play Activities
◆ Story‐Based Activities
◆ Mindfulness‐Based Activities
◆ Academic Activities

By examining these specific theoretical frameworks and activity types, we aim to shed light on potential associations that may explain the variability in self‐regulation outcomes among preschool children. This approach will enable us to make more informed conclusions about the association between different types of activities, theoretical foundations, and self‐regulation.

#### 1.3.3 Instructional method

The development of self‐regulation theories leans on an amalgamation of classical information processing theory and constructivism, thus making room for either perspective in shaping instructional methods. The classical view likens the human mind to a computer, with a spotlight on knowledge transfer via teacher‐centered, didactic instruction such as teacher‐led questioning, explanations, and feedback to students (Reynolds & Miller, 2003; Schunk & Zimmerman, 2003). On the other hand, constructivism accentuates knowledge construction through student‐led, challenging, and engaging discovery learning and problem‐solving activities (Reynolds & Miller, 2003; Schunk & Zimmerman, 2003). Extensive empirical evidence underpins the efficacy of a constructivist instructional approach (Barker et al., 2014; Krafft & Berk, 1998; Whitebread & O'Sullivan, 2012).

Several meta‐analyses support this view, illustrating the relative advantages of instructional approaches grounded in social‐cognitive learning theories over those based on metacognitive or motivational learning theories for primary and secondary students (Dignath et al., 2008; Dignath & Büttner, 2008). Interestingly, Hattie's meta‐meta‐analysis suggests that the role of the teacher as an activator promotes learning, autonomy, and self‐regulation more effectively than the teacher as a facilitator (Hattie, 2009). Notably, some pedagogical characteristics listed by Hattie do not clearly align with the dichotomy of direct and constructivist instructional approaches, necessitating further investigation.

A case in point is “Feedback,” which is considered a direct instructional method and a characteristic of a teacher as an activator, but it can also be obtained from students or self‐generated during self‐guided discovery (Hadwin, 2008). Likewise, individualized instruction, often seen as a feature of the teacher as a facilitator, can also be viewed as a direct method for strategy instruction that students can model and practice.

The instructional context also influences the development of self‐regulation. It is widely accepted that continuous exposure to age‐appropriate tasks that gradually increase in complexity can enhance self‐regulation and executive function beyond children's current capacities (Diamond, 2011; Diamond & Lee, 2011; Hadwin, 2008). This is evidenced by Fernyhough and Fradley (2005), which observed higher rates of self‐regulatory private speech in preschool children as the complexity of the task increased, despite no predictive link to future task performance.

Furthermore, the level of instructional support and scaffolding may need to be reduced over time to promote student autonomy (Hadwin, 2008). However, Pakarinen et al. (2011) found a negative correlation between instructional support and task avoidance in Finnish kindergarten children suggesting that reducing support might negatively affect students during complex tasks.

Lastly, the role of rewards in promoting self‐regulation has elicited mixed findings. While performance‐contingent and engagement‐contingent rewards have been found to reinforce positive self‐regulatory behaviors (Martinez‐pons, 2010; Schunk, 1983, 1984; Selart et al., 2008), they can simultaneously dampen creativity (Selart et al., 2008). In contrast, Joussemet et al. (2004) found that promoting autonomy yielded better self‐regulation results than rewards in primary school children, although the sample and measurement methods differed from preschool populations.

To inform and potentially refine our Theory of Change, we will delve into the nuanced dynamics of instructional methods. Specifically, we will investigate the following characteristics under the category of Instructional Method:

◆ Role of Instructor: Whether the intervention was delivered by preschool teachers as opposed to research assistants or outside experts
◆ Method of Instruction: Whether a direct or constructivist method of instruction was employed
◆ Type of Feedback: Whether students received attributional feedback or progress feedback as part of the intervention
◆ Fading of Instructional Support: Whether instructional support was gradually reduced over the course of the intervention
◆ Task Complexity Adjustment: Whether the difficulty of the tasks was adjusted to the student, such as a gradual increase in task complexity
◆ Performance‐Based Rewards: Whether students were rewarded based on their performance
◆ Engagement‐Based Rewards: Whether students were rewarded based on their engagement

By examining these characteristics, we aim to extend our understanding of the potential mechanisms that could influence variations in self‐regulation outcomes. This exploration is intended to contribute to the mapping of intervention characteristics that may be associated with more effective outcomes.

#### 1.3.4 Academic skills as the secondary dependent variable

A robust body of literature consistently supports the notion that self‐regulation plays a pivotal role in academic achievement (Blair, 2002; Blair & Diamond, 2008; Blair & Raver, 2015; Borkowski & Thorpe, 1994; Braund & Timmons, 2021; Joussemet et al., 2004; McClelland et al., 2019). This consensus is grounded in the observation that children with well‐developed self‐regulation tend to display a range of behaviors that facilitate learning. These behaviors include the ability to follow instructions, effectively utilize learning resources, form positive relationships, resist distractions, and persevere through challenges. Consequently, such children are often more adaptable across various settings, including educational environments (Braund & Timmons, 2021; Perry et al., 2018).

Several studies have underscored the importance of self‐regulation in the foundational stages of academic development, particularly in literacy and math (Blair & Razza, 2007; G. J. Duncan et al., 2007; Gestsdottir et al., 2014; Howse et al., 2010; Korucu et al., 2022; Lonigan et al., 2022; McClelland, Cameron, Connor, et al., 2007; Sawyer et al., 2015; von Suchodoletz et al., 2009, 2013). However, the role of executive function, a specific aspect of self‐regulation that includes working memory, in these academic skills is less clear. For instance, Korucu et al. (2022) found a correlation between general self‐regulation, executive function, and pre‐academic skills but did not find the same for emotion regulation. Similarly, Distefano et al. (2021) observed that while executive function abilities relate to literacy and numeracy, they did not significantly impact when considered alongside other aspects of self‐regulation. This nuanced relationship is further complicated by ambiguous findings regarding the causal link between working memory and academic skills (Melby‐Lervåg et al., 2013; Melby‐Lervåg & Hulme, 2016). These intricacies suggest that while self‐regulation is undeniably crucial for academic development, the specific contributions of executive function warrant further exploration.

Meta‐analytic reviews generally indicate that self‐regulation interventions positively influence literacy and mathematical skills, despite differences in the demographic groups studied compared to our review (Dignath et al., 2008; Dignath & Büttner, 2008; Hattie et al., 1996; Pandey et al., 2018; Takacs & Kassai, 2019). For instance, Baron et al.'s (2017) meta‐analysis of Tools of the Mind interventions—specifically designed for preschoolers, reveals some uncertainty about the reliability of these findings.

Some scholars, such as R. Jacob and Parkinson, have critiqued the existing body of self‐regulation interventions, pointing to weak causal evidence of a relationship between self‐regulation and academic achievement. This skepticism is partly attributed to methodological limitations, including insufficient control for confounders and the existence of potential moderators influencing the effect of the intervention (R. Jacob & Parkinson, 2015). Tominey and McClelland (2011) provide a notable example, demonstrating the positive effects of a self‐regulation intervention on preschoolers' academic skills. However, it leaves an opportunity for further investigation by not delving into the underlying mechanisms via moderation or mediation analysis. This common gap in the literature underscores the need for a deeper understanding of these causal relationships and the role of potential moderators and mediators.

Preschool‐based interventions targeting self‐regulation could also have a direct impact on academic skills. Activities that require working memory, inhibition, and cognitive flexibility could improve self‐regulation, literacy, and mathematical skills. Pretend play, often associated with storytelling and making up narratives, is inherently linked to literacy skills (Braund & Timmons, 2021). Certain academic activities could directly improve academic skills without a moderating effect of self‐regulation (Lonigan et al., 2022). Through these cognitive challenges and enriching learning experiences, interventions could simultaneously promote self‐regulation and academic skills.

While our study does not aim to establish causality, it seeks to critically assess whether preschool‐based interventions that promote self‐regulation are associated with improvements in academic skills. In alignment with the existing literature, we define these academic skills as:

◆ Literacy skills
◆ Math skills

**Figure 1:**
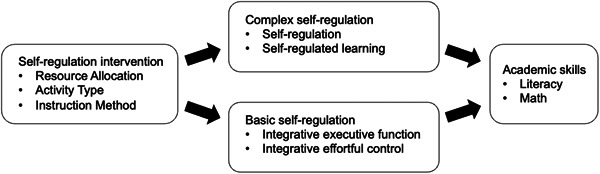
A Theory of Change logic model illustrating how self‐regulation interventions influence self‐regulation, which in turn is associated with academic skills. Arrows represent influence or association, not direct causality.

By specifying these components, we aim to provide a focused framework for assessing the association between self‐regulation interventions and academic skills in preschool settings (see Figure 1).

### 1.4 Why it is important to do this review

Several systematic reviews have explored the effects of self‐regulation interventions or related approaches (e.g., Baron et al., 2017). However, there is a gap in the literature when it comes to examining the specific characteristics that make interventions targeting children in preschool settings.

A recent Campbell review by Baron et al. (2017) uniquely focused on *Tools of the Mind*, a comprehensive curriculum for early childhood education. Pandey et al. (2018) conducted a meta‐analytic synthesis of 50 universal self‐regulation interventions for children and adolescents. Their review primarily centered on multi‐component interventions, which include curriculum‐based programs and personal and social skills training, as well as interventions not initially intended for self‐regulation enhancement, such as yoga and mindfulness. The team applied rigorous selection criteria, focusing solely on randomized controlled trials that explicitly mentioned self‐regulation. This strict approach led them to identify just one preschool intervention that used martial arts to foster self‐regulation.

Additionally, four meta‐analyses have summarized the evidence for school‐based interventions on self‐regulated learning, examining how effects varied with training characteristics. These reviews included a range of age groups, from preschoolers to secondary school students (Dignath et al., 2008; Dignath & Büttner, 2008; Hattie et al., 1996; Wang & Sperling, 2020). While they did cover multiple age groups, none specifically focused on preschool‐based interventions, which is the primary concern of our review.

Our systematic review builds upon Day et al. (2022), who also investigated the qualities of effective preschool self‐regulation interventions. It is worth noting some limitations in their review, such as the exclusion of gray literature, which could offer practical insights and counteract publication bias. They also concentrated on interventions rooted in Self‐Determination Theory and provided a narrative summary rather than a quantitative synthesis of the data.

Lastly, two reviews conducted moderator analyses to explore the characteristics of interventions. Scionti et al. (2020) assessed the impact of cognitive training interventions on executive functions in preschoolers, while Takacs and Kassai (2019) focused on interventions that enhance executive function abilities in children aged two to 12 years. However, these studies targeted different demographics and facets of self‐regulation than those examined in this review.

To the best of our current understanding, no meta‐analysis has been conducted that specifically examines the effects of preschool‐based interventions aimed at promoting both basic and complex self‐regulation.

## 2 OBJECTIVES

The aim of this systematic review is to advance our understanding of the key characteristics of effective preschool‐based interventions designed to foster self‐regulation. To accomplish this, the review addresses the following questions:

1. What types of preschool‐based interventions have been developed to promote self‐regulation?
2. What is the average effect of these preschool‐based interventions on self‐regulation, focusing on four key constructs: integrative effortful control, integrative executive function, self‐regulation, and self‐regulated learning?
3. What characteristics—such as Resource Allocation, Activity Type, and Instruction Method—could potentially contribute to the effects of preschool‐based interventions in promoting self‐regulation?

## 3 METHODS

### 3.1 Criteria for considering studies for this review

#### 3.1.1 Types of studies

##### Years considered

We will not exclude studies by year of publication.

##### Language

We will include studies that were written in English.

We will exclude studies written in languages other than English.

##### Publication status

We will include empirical studies that report primary data obtained first‐hand through the data collection (Sindin, 2018). Eligible studies may be published (e.g., journal articles, book chapters, conference proceedings) or unpublished (e.g., dissertations) literature.

We will exclude reviews, conceptual papers, introductory book chapters, or other sources that do not contain primary data.

##### Study designs

We will include the following interventional study designs that allow for causal inference:

1. Randomized Controlled Trial (RCT):
  a. Standard (parallel) RCTs
  b. Cluster‐RCTs
  c. Crossover RCTs
2. Non‐Randomized controlled Studies of Intervention (NRSI):
  a. Quasi‐RCTs
  b. Non‐RCTs

We consider randomized controlled trials (i.e., RCTs), in which units are randomly assigned to an intervention (treatment) group, a comparison group, or a control (business‐as‐usual) group, to be the optimal study design for obtaining unbiased estimates of intervention effects (Reeves et al., 2023). The difference between standard and cluster‐RCTs lies in the unit of randomization. Standard RCTs use individuals as the unit of randomization, whereas cluster‐RCTs use groups of individuals as the unit of randomization. Crossover RCTs also use randomization, although the initial group assignment is switched mid‐study so that the same participants undergo both intervention and control conditions in two consecutive phases. The strength of crossover RCTs is their efficiency. Compared to standard RCTs with a simple parallel‐group design, crossover RCTs require fewer participants because each participant acts as their own control group (J. P. T. Higgins, Eldridge, et al., 2023). However, crossover RCTs may not be suitable for self‐regulation interventions as there may be carry‐over effects between phases, which we will avoid by extracting data only from the first phase.

Non‐randomized controlled studies of intervention (i.e., NRSIs) inherently carry a greater risk of bias (Ferriter & Huband, 2005; J. A. Sterne et al., 2023). However, we have opted to include NRSIs in our review for several reasons. First, due to the limited number of available RCTs, incorporating NRSIs can enrich our understanding of the current state of evidence concerning self‐regulation interventions. Second, high‐quality NRSIs can approximate the rigor of RCTs in certain contexts (Ferriter & Huband, 2005). Additionally, NRSIs often offer greater external validity, allowing for broader generalization of the findings to real‐world settings. Among NRSIs, two study designs are considered particularly relevant: quasi‐RCTs and non‐RCTs (Cochrane Effective Practice and Organisation of Care, 2017; Reeves et al., 2017, 2023). In both of these designs, control over participant allocation is in the hands of the investigator. Quasi‐RCTs employ a quasi‐random method of allocation (e.g., based on participants’ birthdays), while non‐RCTs use a non‐random method. There is some debate in the literature about whether controlled before‐after studies (i.e., CBA studies) should be distinguished from non‐RCTs. CBA studies do not involve active group assignments by researchers (W.‐P. Schmidt, 2017). However, some researchers, such as Polus et al. (2017), argue that this distinction is artificial and impractical, often due to poor reporting. In light of this, we will consider specific study design features when assessing the risk of bias but will not make a distinction between CBAs and non‐RCTs.

We will exclude study designs that use difference‐in‐differences analyses and interrupted time series, as these methods are most commonly used in natural experiments where interventions can be explored but are not under the investigator's control (Craig et al., 2012; Polus et al., 2017). Our focus is on controlled experiments where the investigator designs, implements, and evaluates interventions targeting children's self‐regulation in the preschool classroom. We will also exclude studies that use instrumental variables and regression discontinuity for the same reason. These methods reflect the treatment effect only for a subgroup of the population, not everyone in the sample, and are known to produce larger estimates than the intention‐to‐treat approach (Angrist, 2006), which we will address in this review. Furthermore, we will exclude other intervention studies that do not control for confounding factors (e.g., uncontrolled before‐after studies), use mediation, latent growth, or cross‐lagged analyses without reporting pre‐and post‐intervention outcomes for the intervention and control groups, or use only a qualitative method of data collection and analysis (Noyes et al., 2023). Finally, we will exclude observational studies, such as cross‐sectional studies, or other studies that do not assess the effects of interventions on child outcomes.

#### 3.1.2 Types of participants

We will include studies that target typically developing preschool‐aged children between the ages of three and six regardless of gender, ethnicity, language learning status, socioeconomic status, and other demographic risk factors (see OECD, 2022). When we find interventions that include both the target population (e.g., preschool‐aged children) and the nontarget population (e.g., school‐aged children) without reporting separate statistics for the two groups, we will attempt to contact the authors of the studies to obtain relevant data on the target population. Despite these efforts, it may be impossible to reach the study authors—in which case we may still choose to include these studies if the students’ backgrounds (see confounding factors in the section “Risk of bias in individual studies”) are sufficiently similar and relevant to interventions in real‐world contexts that often involve both preschoolers and first graders. We anticipate that this approach will increase the ecological validity of the meta‐analysis results. Thus, if it is difficult to obtain data only from preschoolers, we will still include the data as long as we find sufficiently similar baseline characteristics in preschoolers and other children.

We will exclude children with behavioral or socio‐emotional problems (e.g., externalizing problems) or children at risk for a medical, cognitive, behavioral, or learning disorder (e.g., attention deficit hyperactivity disorder, autism spectrum disorder). Because tier two or three interventions often target these children, we will exclude such interventions. However, we anticipate that we will find some studies that do not distinguish between children with and without disabilities. In such cases, we will include studies whose participants are predominantly children with normal development. Although it is difficult to set a cut‐off point, we will justify our decision to exclude and record the proportion of atypically developing children in the included studies.

#### 3.1.3 Types of interventions

##### Interventions

We will include universal or tier‐one interventions that focus primarily on promoting self‐regulation or self‐regulated learning in preschool children. Interventions can be of any duration and can be delivered by either school staff (e.g., preschool or kindergarten teachers) or outside experts (e.g., researchers) as long as the interventions can be readily implemented by teachers with minimal training (McClelland & Cameron, 2012; Zhou et al., 2012). For example, extensive mindfulness or music practice requires teachers with such expertise, so we will exclude these interventions. In addition, we will look for direct interventions in the form of tasks or activities that are specifically designed to improve children's self‐regulation, while teachers can be trained to effectively implement the intervention. Interventions may also target other outcomes of interest, but the focus must be on self‐regulation. Moreover, we will include interventions that target executive function in relation to this criterion. This is because some interventions targeting executive function (1) train not only discrete components of executive function, but also self‐regulation (e.g., integrative executive function), and (2) include relevant measures of self‐regulation.

We will exclude interventions unrelated to school activities, such as self‐regulation of eating or health behaviors, interventions that require expertise and extensive training (e.g., occupational therapy), and prepackaged interventions that were not originally intended to promote self‐regulation, including contemplative practices (e.g., mindfulness and meditation; Flook et al., 2015), sports (e.g., martial arts; Lakes & Hoyt, 2004), music (Shen et al., 2019), literacy (Cavanaugh et al., 2017), or mathematics (DeFlorio et al., 2019). Nevertheless, we will include interventions that selectively incorporate some elements of such practices (e.g., academic tasks or mindfulness, musical, or physical activities) into activities primarily designed to promote self‐regulation.

Moreover, to focus on the sources of heterogeneity according to intervention characteristics of interest (i.e., Resource Allocation, Activity Type, and Instructional Method), we will exclude preschool‐based interventions that aim to indirectly influence children's social environment by creating a favorable classroom climate (e.g., the *Conscious Discipline* program), the teacher‐child relationship (e.g., the *Chicago School Readiness Project*), and professional development or parent training to improve regular classroom practice or child‐rearing (that goes beyond the training required to implement the intervention; e.g., the *Research‐based Developmentally Informed Parent* program or *REDI‐P*).

Finally, we will exclude complex interventions such as interventions that combine direct and indirect causal pathways to develop self‐regulation (i.e., a combination of child, teacher, and/or parent training) or interventions that are integrated into (and thus inseparable from) school curricula (e.g., *Tools of the Mind*). Although *Tools of the Mind* focuses on developing self‐regulation through structured dramatic make‐believe play, the program takes a holistic approach to promoting multiple domains of child development (e.g., academic skills and socio‐emotional development, including self‐regulation) as a comprehensive curriculum (Baron et al., 2017, 2020; Bierman & Torres, 2015). Therefore, the effectiveness of the *Tools* curriculum depends on nonlinear interactions between the key components of *Tools* and the context under study. The complexity of the intervention makes it difficult to attribute observed effects to characteristics of the intervention (N. C. Campbell et al., 2007; Craig et al., 2008; Pigott & Shepperd, 2013). For the same reason, we will exclude other existing educational programs or curricula such as *Montessori* education (Ervin et al., 2010; Lillard, 2012; Lillard et al., 2017), the *Promoting Alternative Thinking Strategies* (*PATHS*) curriculum (Morris et al., 2014), the *Head Start Research‐based, Developmentally Informed* (*REDI*) intervention (Bierman et al., 2008, 2014), the *Chicago School Readiness Project* (*CSRP*; Jones et al., 2013; Raver et al., 2011), and *Conscious Discipline* (K. L. Anderson et al., 2020). Similarly, we will exclude interventions that target self‐regulation as part of a broader set of abilities (e.g., school readiness, socio‐emotional skills, critical thinking, understanding and expressing emotions, and Theory of Mind), although this decision often requires a review of the full text to confirm what the authors mean by these terms.

##### Setting

We will include interventions that take place in preschools, defined as formal out‐of‐home education and care that children attend before entering primary school (Dietrichson et al., 2020). Preschools may also be referred to as pre‐primary schools, play schools, kindergartens, nursery schools, daycare centers, and pre‐kindergartens. Note that some preschool programs may be housed on primary school campuses. Although we will not exclude preschool‐based interventions that are combined with another intervention outside of the school setting under this criterion (e.g., parent training at home), we will exclude complex interventions under the criterion for interventions.

We will exclude interventions that take place entirely outside the preschool setting (e.g., foster care, nannies, or parent training at home), except for summer programs to prepare children for kindergarten whose target population and intervention characteristics are sufficiently similar to the preschool‐based interventions we examine. We will also exclude computer‐mediated interventions (e.g., training that incorporates information and communication technology such as computers or tablet apps).

#### 3.1.4 Types of outcome measures

We will include studies with primary outcome measures that assess self‐regulation. We will not exclude studies based on a secondary outcome or duration of follow‐up.

##### Primary outcomes

We will include measures of the complex self‐regulatory processes: self‐regulation and self‐regulated learning. Measures of self‐regulation include performance‐based measures such as the Preschool Self‐regulation Assessment (PSRA; Smith‐Donald et al., 2007) and the Preschool Situational Self‐Regulation Toolkit (PRSIST) assessment (Howard et al., 2019) or questionnaires such as the Child Behavior Rating Scale (CBRS; Bronson et al., 1990) and the Child Self‐Regulation and Behaviour Questionnaire (Howard & Melhuish, 2017). Self‐regulated learning (i.e., self‐regulation of learning) can be assessed through various phases of problem‐solving tasks (e.g., planning, monitoring, and evaluating) using the C. Ind. Le Coding Framework (i.e., the observational coding framework for verbal and non‐verbal indicators of metacognitive and self‐regulatory processes in children aged three to five; Whitebread, Coltman, Pasternak, et al., 2009), the Children's Independent Learning Development checklist (CHILD 3‐5; Whitebread, Coltman, Pasternak, et al., 2009), and the Train Track Task, which captures a metacognitive aspect of self‐regulated learning (Bryce et al., 2012; Bryce & Whitebread, 2015).

In addition, we will include performance‐based measures of basic self‐regulatory processes: integrative executive function and integrative effortful control. More specifically, we are interested in measures that assess the active integration of components of executive function (i.e., working memory, inhibition, and shifting; Hofmann et al., 2012; McClelland, Cameron, Connor, et al., 2007; McClelland, Cameron, Wanless, et al., 2007; Rueda et al., 2005) or effortful control (i.e., delaying gratification, gross motor control, fine motor control, suppress‐initiate response to signals, and effortful attention; Kochanska & Knaack, 2003; Murray & Kochanska, 2002; Zhou et al., 2012) within a task. For executive function measures, we will include Heads‐Toes‐ Knees‐Shoulders (McClelland & Cameron, 2012), the Hearts and Flowers task (Wright & Diamond, 2014), the Dots test (or task; Davidson et al., 2006; Diamond et al., 2007), and the Minnesota Executive Function Scale (Carlson & Zelazo, 2014), which require children to pay attention, use working memory to remember the instruction, and use inhibitory control to respond to the task, despite potential ecological validity issues (Hammer, 2018). Because our previous knowledge of such measures is limited, particularly for integrative effortful control skills, we will include other measures that meet this criterion.

Accordingly, we will exclude studies that do not measure basic and complex self‐regulatory processes at the child level. In addition, we will exclude measures of discrete components of executive function or effortful control (McClelland & Cameron, 2012) or measures of discrete executive functions that are grouped together as global executive function. These include the Behavior Rating Inventory of Executive Function—Preschool Version (Sherman & Brooks, 2010), the Early Years Toolbox (Howard & Melhuish, 2017), and the NIH Toolbox Cognition Battery (Zelazo et al., 2013).

##### Secondary outcomes

Secondary outcomes include all quantitative measures of academic skills (i.e., emergent literacy and math skills).

### 3.2 Search methods for identification of studies

#### 3.2.1 Electronic searches

**Figure 2:**
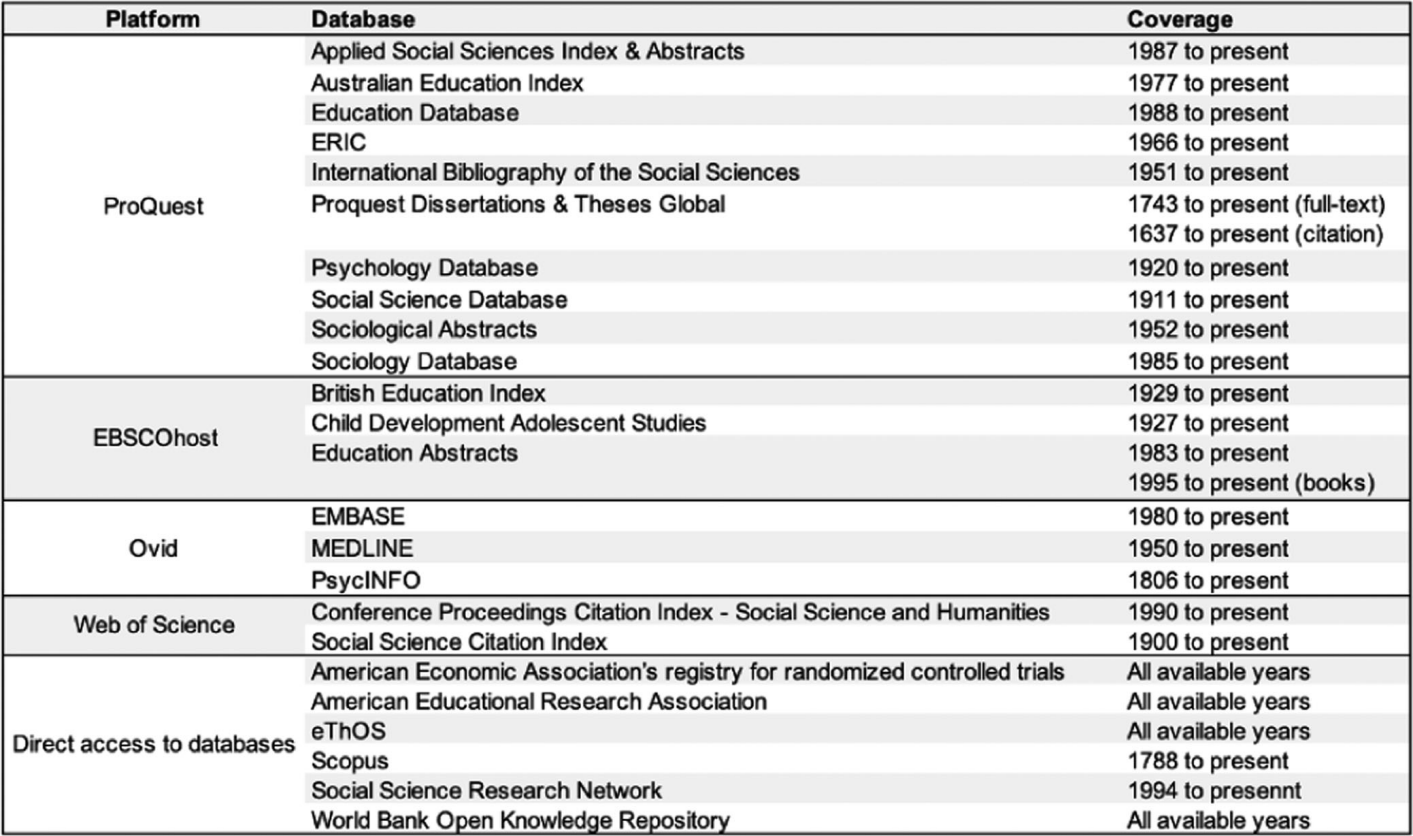
Databases.

We determined the databases following the list of databases in the Campbell Searching for Studies Guide (Kugley et al., 2017) after consulting with an information retrieval specialist (the 15th reviewer). Accordingly, we will search the following electronic databases, some of which index gray literature such as conference proceedings, theses, and dissertations (see Figure 2). Nonetheless, we will exclude reports from governments, non‐governmental organizations, and think tanks whose interest typically lies in pragmatic trials of complex interventions, as our preliminary search yielded only complex interventions that are outside the scope of this review.

The following search terms are ordered according to the PICO framework using the Boolean operators OR and AND to achieve high sensitivity within concepts (see Supporting Information: Appendix 2). We will not include acronyms in the Boolean logic, as we expect to capture the fully spelled version. Moreover, we will not use proximity operators because we did not find any new results when they were added to the search. Although terms such as emotion regulation/control, behavior regulation/control, self‐management, or metacognition are theoretically linked to the construct of self‐regulation that we are interested in, the constructs do not neatly overlap (see the section “Description of the condition”). Therefore, we will not include these terms in the Boolean logic to ensure a balance between sensitivity and specificity.

Prior to finalizing this search strategy, a pilot search was conducted by AK and KS to test the efficacy of the search terms and Boolean operators. The insights gained from this pilot search were instrumental in shaping the final search strategy. KS, an information retrieval specialist, will oversee the literature search, while AK will be responsible for exporting the search results in either XML or RIS file formats. The results will then be deduplicated to ensure the quality and relevance of the literature included in the review.

#### 3.2.2 Searching other resources

To supplement the electronic search, we will manually search Google and the websites we selected for their potential to find relevant gray literature (i.e., Brookings Institution, National Education Association, National Institute for Early Education Research, and The Economic and Social Research Institute) using keywords and search filters. Moreover, we will search the reference list of relevant reviews (e.g., articles and book chapters), tables of contents of relevant journals (e.g., *Child Development*, *Early Child Development and Care*, *Early Childhood Education Journal*, *Early Childhood Research Quarterly*, *Early Education & Development*, *Frontiers in Psychology*, *International Journal of Behavioral Development*, *Journal of Early Childhood Research*, and *Science*) and conference proceedings (e.g., *Advances in Cognitive Psychology Conference*, *Applied Cognitive Psychology Conference*, *British Psychological Society Conference*, *Cognitive Development and Social Cognition Conference*, *Developmental Psychology and Cognitive Development Conference*, *Developmental Psychology and Cognitive Development Conference*, *Human Development Conference*, *Memory and Cognition Conference*, *Society for Research into Child Development Conference*, *Society for Research on Educational Effectiveness Conference*, and *Theories of Cognitive Development Conference*) between 2005 and 2022. The above journals and conference websites were selected based on the potentially relevant studies found in our preliminary search results, although the list is not exhaustive and may change depending on the search results. In addition, we will perform a backward citation search by checking the reference lists of included studies and a forward citation search by examining the studies associated with the included studies on Scopus, the Web of Science, and Google Scholar. Finally, we will provide the inclusion criteria and a list of included studies to study authors and other experts via email to ask if they know of additional published or unpublished studies that can be added to this review (Kugley et al., 2017). We will update the search toward the end of this review.

### 3.3 Data collection and analysis

#### 3.3.1 Selection of studies

**Figure 3:**
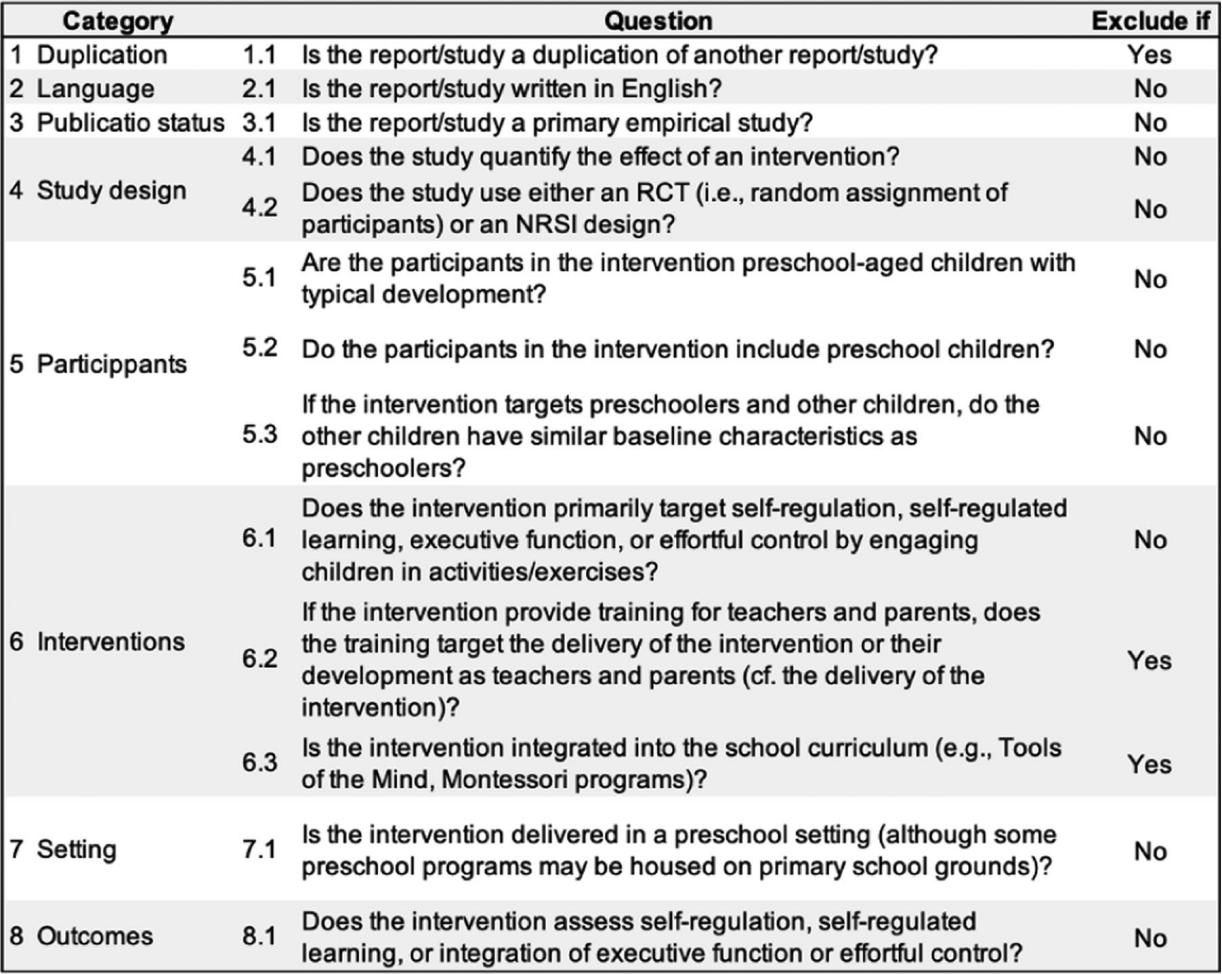
Screening questions.

We will upload the results of the literature search into Covidence, a web‐based software program designed to facilitate de‐duplication, study selection, and collaboration among reviewers. The first reviewer has formulated screening questions based on the eligibility criteria (see Figure 3). To ensure the effectiveness and clarity of these screening questions, a pilot test will be conducted on a sample of 10 to 20 reports. This range allows for flexibility and ensures a more representative sample for refining our screening approach.

After the pilot test, the screening questions will be reviewed and finalized in consultation with IS, MM, KES, and ECY. If the initial 10 reports provide sufficient insights, we may proceed to the main review. However, if issues arise or further refinement is needed, we may extend the pilot to include up to 20 reports. Once the screening questions are finalized, they will be shared among all reviewers to facilitate the study selection process.

Studies are selected in two stages. The first screening (title and abstract) will exclude obviously irrelevant reports to save time. The second screening (full text) will be used to further exclude irrelevant studies based on a more detailed review of the full texts.

To ensure the reliability of the study selection, each report or study will undergo a two‐round screening process. The first round will be conducted by AK, who will screen all titles, abstracts, and full texts. Given that this review is taking place over an extended period, it is not feasible to specify the exact number of reviewers for the second round. The number will depend on reviewer availability at the time of each screening phase and will be determined through discussion with each potential reviewer.

Prior to screening, only those reviewers assigned to this task will undergo training. The training will include viewing instructional videos created by AK, participating in hands‐on exercises, and engaging in discussions to clarify any ambiguities regarding screening procedures.

For the first screening, AK will screen all titles and abstracts of the initial sample, while other available reviewers will independently screen their assigned portion of titles and abstracts. If the title and abstracts do not contain enough information to determine eligibility, the reports will be included for further review. For the second screening, AK will screen all the full texts of the potentially included studies, whereas other available reviewers will independently review their allocated number of full texts.

Given the extended timeline and potential variability in reviewer availability, we will not calculate inter‐rater reliability. Instead, any disagreements between the first and second rounds of screening will be resolved through discussion to ensure the validity of the selection process.

#### 3.3.2 Data extraction and management

##### Data collection process

AK has developed a comprehensive set of coding instructions for both the study level and the effect size level to guide the data extraction process (see Supporting Information: Appendix 3 for coding instructions). These instructions were tested and subsequently refined in consultation with IS. This approach aims to ensure consistency and transparency among reviewers during data extraction, thereby minimizing the need for frequent reference to original data sources during both data synthesis and risk of bias assessment (Li et al., 2023).

For data collection, two reviewers will be assigned to extract data from each eligible study. Due to the review's extended timeline and varying reviewer availability, we cannot specify the exact number of reviewers for this phase. Only those reviewers assigned to this task will undergo training, which includes watching an instructional video, participating in calibration exercises, and engaging in discussions to resolve any ambiguities related to data collection procedures.

AK will take the lead in data extraction, using a standardized grid to collect data from all included studies. The role of the other reviewers will involve verifying the accuracy of AK's study‐level coding and independently performing effect size‐level coding for their allocated sections of the included studies.

Any disagreements that arise during this process will be resolved through discussion among the reviewers. If additional clarification is needed, we will not hesitate to contact the authors of the studies in question.

We will extract the following study characteristics:

##### Study‐level coding

1. Bibliographic information: date of extraction; report ID; study ID; publication type; author; publication year; study title
2. Study design: sampling method; duration of enrolment; design type; statistical method used to estimate the intervention effect; statistical method used to control for covariates (see Supporting Information: Appendix 1 for the review of covariates relevant to self‐regulation development), clustering, and missing data
3. Participants: age; gender ethnicity; socioeconomic status; language learning status; country (countries)
4. Intervention:
  a. Short narrative description of the intervention
  b. Conceptual framework: self‐regulation concept (construct and definition); self‐regulation theory (model); self‐regulation system; domain specificity
  c. Training characteristics: Resource Allocation (period, volume, duration, frequency, adherence, class size, number of adults in one classroom, pupil‐teacher ratio); Activity Type (self‐regulation‐theory‐ based activities, physical activities, musical activities, pretend play activities, construction play activities, story‐based activities, mindfulness‐based activities, or academic activities); Instructional Method (routes of delivery, learning theory, feedback on performance, fading of instructional support, adapted task difficulty, rewards)
5. Miscellaneous: main conclusions; reference to other relevant studies; need for clarification; other comments

##### Effect size‐level coding

1. Outcome:
  a. Self‐regulation: construct; self‐regulation system; domain specificity; the name of the measurement; measurement type
  b. Academic skills: construct; measurement
2. Data: original metric; aggregation method; time points of assessment; covariates; clustering; missing data
3. Results: sample size; means; standard deviations; effect estimates; standard errors; intention‐to‐treat or per‐protocol effect
4. Additional questions for crossover RCTs

##### Primary outcomes

We will differentiate outcomes in self‐regulation based on their operational definitions and types of measurement.

First, we will consider four distinct approaches to conceptualizing child self‐regulation: these include self‐regulation, self‐regulated learning, executive function, and effortful control. While executive function has traditionally been the focus of cognitive neuroscience and clinical psychology, primarily in contexts devoid of emotional influence, effortful control has been examined within the realm of temperament research, particularly in emotionally charged settings (Zhou et al., 2012). Despite these divergent research traditions, Zhou and colleagues highlight several areas where the definitions and operational aspects of executive function and effortful control overlap. They advocate for a unified model that integrates these two theoretical frameworks.

Second, we will categorize self‐regulation into two primary systems: the cognitive (“cool”) system, which focuses on cognition and behavior, and the affective (“hot”) system, which centers on motivation and emotion (Dent, 2013; Zhou et al., 2012). Additionally, we will consider an integrated approach that combines both cool and hot dimensions of self‐regulation. Prior research has established a connection between the development of executive function and cool self‐regulation with academic achievement, while effortful control and hot self‐regulation have been associated with socio‐emotional development (McClelland & Cameron, 2012; Willoughby et al., 2011; Zhou et al., 2012).

Third, we will differentiate between domain‐general self‐regulation, which refers to foundational abilities applicable across various life contexts, and domain‐specific self‐regulation, which focuses on abilities tailored to particular settings or subjects such as academics or social interactions (Gunzenhauser & Saalbach, 2020). This distinction is vital for assessing the scope and applicability of self‐regulatory interventions, as it allows us to understand whether the abilities developed are broadly transferable or more targeted within specific domains.

Fourth, we will distinguish between online and offline measures of self‐regulation based on the timing of the data collection (Araka et al., 2020; L. Jacob et al., 2019; McClelland & Cameron, 2012; Rovers et al., 2019; Schmitt et al., 2015). Online measures collect data during the execution of the actual learning task, whereas offline measures collect data before and after performance. Specifically, online measures tend to assess ongoing specific self‐regulatory behaviors or strategies as events or states, whereas offline measures are more inclined to assess children's self‐regulation as aptitude or traits or global use of strategies through reflection (Inzlicht et al., 2021, p. 20; Rovers et al., 2019; Winne, 2010).

Fifth, researchers have increasingly noted the ecological validity of direct measures of self‐regulation across contexts compared to indirect measures such as teacher reports or classroom observations (McClelland et al., 2012; McClelland & Cameron, 2015; Schmitt et al., 2015). Overall, we expect that measures that rely heavily on preschoolers’ verbal skills or reflection including thinking aloud, self‐reports, or structured interviews, will be used less frequently (L. Jacob et al., 2019; Whitebread, Coltman, Pasternak, et al., 2009). We plan to examine heterogeneity in the summary effect of the intervention using the four measurement types explained above. In addition, we will use multiple measures of self‐regulation within a single study.

##### Secondary outcomes

We will include all quantitative measures of children's academic skills (e.g., emergent literacy, math skills) as secondary outcomes.

##### Timing of the assessment

We will include data collected during the short‐term (up to five months post‐intervention), medium‐term (six months to 11 months post‐intervention), and long‐term (12 months or more post‐intervention) follow‐up periods as secondary outcomes.

#### 3.3.3 Assessment of risk of bias in included studies

We will assess the potential risk of bias at the level of an individual result (i.e., each estimate of the intervention effect and its variance), focusing on internal validity (i.e., the confidence with which researchers can determine that at least part of the change in the outcome of interest was caused by the intervention; Brewer, 2011; Glasgow et al., 2003; Maul & Katz, 2018). RCTs will be assessed using RoB2 (J. A. C. Sterne et al., 2019), which consists of signaling questions designed to assess five domains of bias (i.e., bias due to the randomization process; bias due to deviations from intended interventions; bias due to missing outcome data; bias in the measurement of the outcome; bias in the selection of the reported result; J. P. T. Higgins, Savović, et al., 2023).

We do not consider the use of simple unrestricted randomization to be appropriate in most RCTs. This is because researchers have warned about the chance imbalances that arise with simple randomization in small trials (Fron Chabouis et al., 2014; Ivers et al., 2012; Kernan et al., 1999). For example, Nguyen et al. (2017) simulated from two previous clinical trials that simple randomization requires at least 1,000 participants to obtain unbiased effect estimates. Therefore, the use of constrained randomization (e.g., pair matching, blocking, stratification, minimization) is highly desirable unless studies include a sufficiently large number of units for randomization.

Similarly, NRSIs will be assessed using the ROBINS‐I tool (J. A. Sterne et al., 2016), which includes six domains of bias (i.e., bias due to confounding; bias in selecting participants for the study; bias in classifying interventions; bias due to deviations from intended interventions; bias due to missing data; bias in measuring the outcome; bias in selecting the reported result; J. A. Sterne et al., 2023). Because successful control of confounding depends on the selection of baseline covariates that might influence the observed intervention effects, we will pay attention to whether studies account for important covariates that are related to child factors (i.e., age; gender; ethnicity; IQ; baseline academic skills; language learning status; baseline self‐regulation), parental factors (i.e., parental education; socioeconomic status; parenting), and environmental factors (i.e., household chaos; media exposure; culture) either by design (in RCTs) or by statistical control (in NRSIs). Because we will exclude intervention studies with co‐interventions based on eligibility criteria, we do not have preliminary considerations for co‐interventions. Nonetheless, we will consider co‐interventions that participants may have received as a potential source of bias.

Two reviewers will independently evaluate each study to achieve a consensus on the final risk‐of‐bias rating. Similar to the procedures for study selection and data collection, the team will consult an instructional video crafted by AK to ensure a standardized approach to assessing the risk of bias. Calibration exercises will be conducted, and any procedural ambiguities will be collaboratively discussed to ensure clarity. Should disagreements arise, they will be resolved through open dialogue. If further clarification is needed, we will reach out to the authors of the respective studies.

AK will take the lead in evaluating all included studies, while additional reviewers will independently assess the segments allocated to them within the pool of selected studies. To facilitate data visualization, we will generate separate graphical representations for RCTs and NRSIs using R, a freely available software for statistical computing and graphics.

#### 3.3.4 Measures of treatment effect

We will use standardized mean differences (*SMD*s) by standardizing the results of individual studies on a uniform scale (i.e., removing variability in measurement scales) before combining them in meta‐analyses. In doing so, we assume that results from different measures assessing the same constructs (e.g., self‐regulation or academic skills) can be combined (J. P. T. Higgins, Li, et al., 2023). In addition, we will use Hedges’ *g* for the *SMD*, which is a bias‐corrected estimator that adjusts for small‐sample bias in Cohen's *d* (Lin & Aloe, 2021). This is because we expect to find some studies with small sample sizes.

Following recent guidance (J. P. T. Higgins, Thomas, et al., 2023; What Works Clearinghouse, 2022; Wilson, 2017), we will either calculate *SMD*s and variances manually from the summary statistics for each intervention group or extract an estimate of the intervention effect directly from a study report:

1. Summary statistics: means; standard deviations; group sample sizes
2. Effect estimates:
  a. effect size; standard error (also computable from a confidence interval, a *z*‐score, or an exact *p*‐value)
  b. unstandardized or standardized regression coefficient; the standard deviation of the dependent variable; group sample sizes; total sample size; a *t* (or *z*) statistic for the regression coefficient (also computable from the standard error or confidence interval of the regression coefficient)

##### Parallel and crossover RCTs

**Figure 4:**
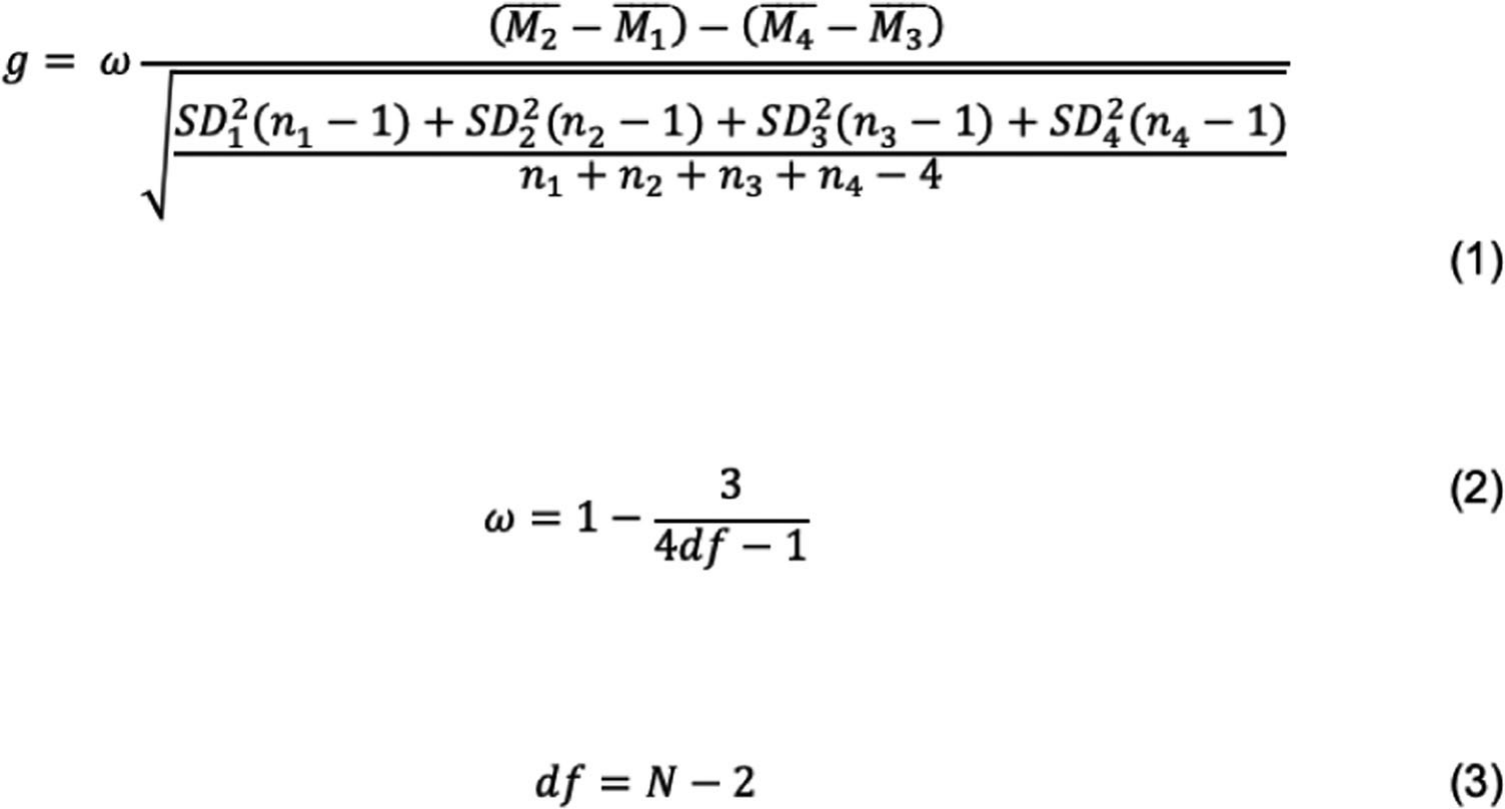
Formulas for calculating Hedges' *g* in RCTs.

For parallel RCTs that use an appropriate randomization method, we will use summary statistics so that intervention effects can be estimated consistently across studies (J. P. T. Higgins, Li, et al., 2023). Similarly, for crossover RCTs, we will use summary statistics from the first trial period (J. P. T. Higgins, Eldridge, et al., 2023). More specifically, we will first calculate Hedges’ *g* from the change‐from‐baseline scores (i.e., the difference between pre‐and post‐intervention scores) for parallel RCTs and crossover RCTs using formula 1 (i.e., a combination of equations 5, 27, 28, and 29 in Wilson, 2017; see Figure 4), where *g* is Hedges’ *g*, *M* is the mean for each group, *SD* is the standard deviation, *n* is the size of each group, *ω* is a small sample size bias correction factor (What Works Clearinghouse, 2022), *df* is the degrees of freedom (What Works Clearinghouse, 2022), *N* is the total sample size, and the subscripts 1, 2, 3, and 4 represent treatment pre‐intervention, treatment post‐intervention (or follow‐up evaluation), control pre‐intervention, and control post‐intervention (or follow‐up evaluation), respectively.

**Figure 5:**
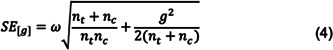
Formula for calculating the standard error of Hedges' *g*.

We will calculate *SE*_*[g]*_ (i.e., the standard error of *g*) using formula 4 (What Works Clearinghouse, 2022; see Figure 5), where *n* is the size of each group and the subscripts *t* and *c* represent the treatment and control groups, respectively.

**Figure 6:**
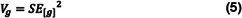
Formula for calculating the variance of Hedges' *g*.

*SE*_*[g]*_ is squared to produce *V*_*g*_ (i.e., the variance of *g*) using formula 5 (see Figure 6).

##### Quasi‐RCTs and non‐RCTs

**Figure 7:**
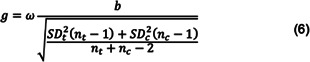
Formula for converting effect sizes to Hedges' *g* in quasi‐RCTs and non‐RCTs.

For quasi‐RCTs and non‐RCTs, we will prioritize extracting adjusted effect estimates to reduce bias due to confounding and missing data (e.g., the Full Information Maximum Likelihood Estimation; Enders, 2001). Therefore, we will attempt to convert available cluster‐adjusted effect sizes (e.g., Cohen's *d*) and variance (e.g., *V*_*d*_, which can also be calculated from the confidence interval) into Hedges’ *g* and its variance (*V*_*g*_) or use unstandardized regression coefficients representing an intervention effect to calculate *g* using formula 6 (What Works Clearinghouse, 2022; see Figure 7), where *g* is Hedges’ *g*, *ω* is a small sample size bias correction factor for individual‐assignment studies, *b* is an unstandardized regression coefficient, *SD* is the standard deviation, and *n* is the size of each group.

**Figure 8:**
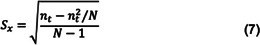
Formula for calculating the standard deviation of the independent variable in quasi‐RCTs and non‐RCTs.

If quasi‐RCTs and non‐RCTs report standardized regression coefficients (*β*), we will transform *β* into *b*. To do this, we will first calculate the standard deviation of the independent variable (*S*_*x*_) using formula 7 (Wilson, 2017; see Figure 8).

**Figure 9:**
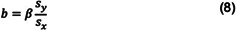
Formula for transforming standardized regression coefficients (*B*) to unstandardized (*b*) in quasi‐RCTs and non‐RCTs.

*S*_*x*_ is used to calculate an unstandardized regression coefficient (*b*) using formula 8 (Wilson, 2017; see Figure 9).

Subsequently, *b* is used to calculate Hedges’ *g* with formula 6.

**Figure 10:**
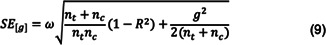
Formula for calculating the standard error of Hedges' *g* using reported *R^2^* in quasi‐RCTs and non‐RCTs.

We will produce *SE*_*[g]*_ for quasi‐RCTs and non‐RCTs in two ways. First, if we can assume that a study's statistical model produced intervention effect estimates that were properly adjusted for covariates, we will use the reported *R* ^*2*^ in calculating *SE*_*[g]*_ using formula 9 (What Works Clearinghouse, 2022; see Figure 10).

**Figure 11:**
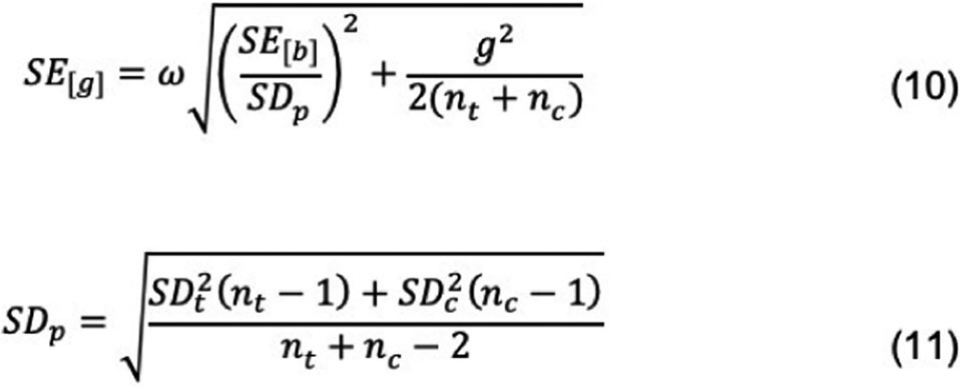
Formulas for rescaling standard error using standard error of unstandardized coefficient and pooled sample standard deviation in studies without reported *R^2^*.

Second, when studies do not report *R* ^*2*^ values, we will rescale the standard error of a correct model using formula 10 (What Works Clearinghouse, 2022; see Figure 11), where we use a standard error (which can also be calculated from a confidence interval) of the unstandardized regression coefficient for an intervention effect (i.e., *SE*_*[b]*_) and a pooled sample standard deviation (i.e., *SDp*; see formula 11) to produce *SE*_*[g]*._

**Figure 12:**
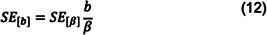
Formula for calculating standard error of unstandardized regression coefficient from standardized coefficient.

When studies report the standard error for a standardized regression coefficient (*SE*_*[β]*_), we will calculate the standard error for the unstandardized regression coefficient (*SE*_*[b]*_) using formula 12 (see Figure 12).

Then, *SE*_*[b]*_ is used to produce *SE*_*[g]*_ with formula 10.

If adjusted effect estimates are not available (and only summary statistics are available) in the study reports, we will calculate Hedges’ *g* and its variance (*V*_*g*_) using formulas 1, 2, 3, 4, and 5, taking into account the potential bias of the calculated effect estimates (see the section “Assessment of risk of bias”). Accordingly, we will record the details of the covariates, with particular attention to those that may act as confounders, and the methods used to account for missing data.

##### Cluster RCTs (and other studies with clustering)

**Figure 13:**
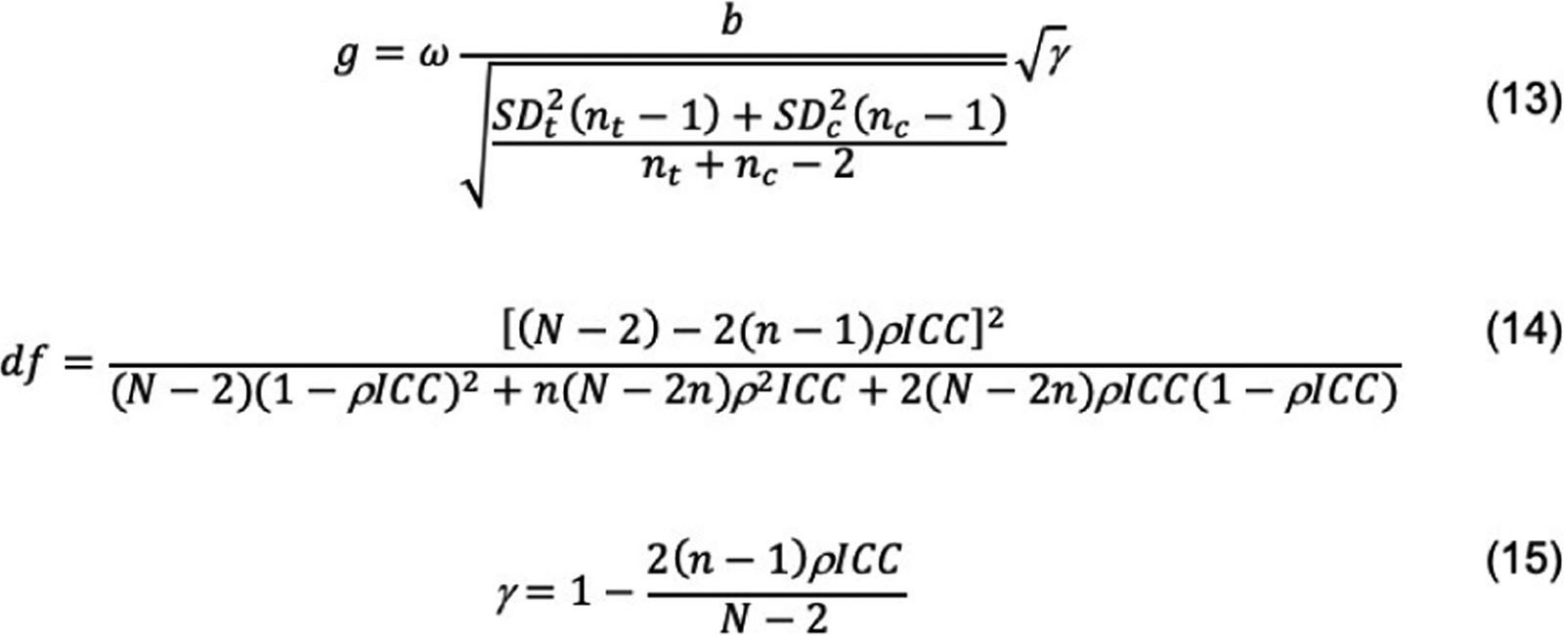
Formulas for adjusting cluster‐RCT effect sizes and variance to Hedges' *g* and its variance, including intraclass correlation adjustment.

Cluster‐RCTs should take into account the clustering of individuals in addition to the biases introduced by confounding and missing data. If study authors properly account for these biases, we will primarily use model‐based effect estimates extracted directly from study reports (typically from a linear regression of cluster‐specific mean outcomes on randomized groups, weighted by cluster size; I. R. White & Thomas, 2016). In other words, we will attempt to convert available cluster‐adjusted effect sizes (e.g., Cohen's *d*) and variance (e.g., *V*_*d*_, which can also be calculated from the confidence interval) into Hedges’ *g* and its variance (*V*_*g*_) or use an unstandardized regression coefficient representing an intervention effect with formulas 2, 13, 14, and 15 (What Works Clearinghouse, 2022; see Figure 13), where *γ* is a small number of clusters adjustment term, *n* is the average cluster size (i.e., the total number of individuals divided by the total number of clusters), and *ρICC* is an intraclass correlation coefficient.

As with *quasi‐RCTs and non‐RCTs*, we will transform standardized regression coefficients (*β*) into unstandardized regression coefficients (*b*) using formulas 7 and 8.

**Figure 14:**
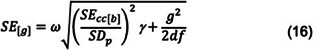
Formula for calculating standard error of Hedges' *g* with cluster‐correction and covariate adjustment.

We will prioritize obtaining standard errors accounting for covariates and clustering in three ways. First, when study authors report a cluster‐corrected standard error (which can also be calculated from a confidence interval) for an unstandardized regression coefficient that represents an intervention effect (*SE*_*cc[b]*_), we will produce *SE*_*[g]*_ using formula 16 (What Works Clearinghouse, 2022; see Figure 14), where *SD*_*p*_ is defined in formula 11, *γ* is defined in formula 15, and *df* is defined in formula 14.

**Figure 15:**
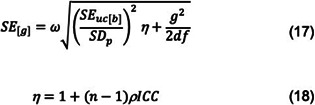
Formulas for adjusting standard errors with design effect in cluster‐RCTs not correcting for clustering.

Second, if cluster‐RCTs account for covariates but not clustering, we will incorporate a design effect term (*η*; see formula 18 for its definition) to adjust standard errors not corrected for clustering (*SE*_*uc[b]*_) using formula 17 (What Works Clearinghouse, 2022; see Figure 15).

If cluster‐RCTs report the standard error for a standardized regression coefficient (i.e., *SE*_*cc[β]*_ or *SE*_*uc[β]*_), we will calculate the standard error for the unstandardized regression coefficient (i.e., *SE*_*cc[b]*_ or *SE*_*uc[b]*_) using formula 12. Then, *SE*_*cc[b]*_ or *SE*_*uc[b]*_ is used to produce *SE*_*[g]*_ with formula 16 or 17, respectively.

**Figure 16:**
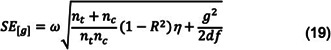
Formula for calculating standard error of Hedges' *g* using *R^2^* and design effect in single‐level model analyses.

Third, if studies report *R^2^* from a single‐level model (e.g., ANCOVA or ordinary least‐squares regression), rather than reporting the standard error of the regression coefficient adjusted for covariates or clustering, we will use *R* ^*2*^ and *η* to produce *SE*_*[g]*_ using formula 19 (What Works Clearinghouse, 2022; see Figure 16).

We will apply the above calculation to quasi‐RCTs and non‐RCTs with clustering of individuals. If adjusted effect estimates are not available in the study reports, we will make full use of the available information to calculate Hedges’ *g* and its variance (*V*_*g*_) adjusted for either confounding, clustering, or missing data. Studies with clustering may not report covariate‐adjusted regression coefficients, but we can calculate Hedges’ *g* (e.g., a combination of formulas 1, 2, 14, and 15) and *V*_*g*_ (e.g., a combination of formulas 2, 4, 14, and 18) adjusted for clustering from the data obtained. As with quasi‐RCTs and non‐RCTs, we will record the details of the covariates, the methods used to account for missing data, and the type and degree of clustering (J. P. T. Higgins, Thomas, et al., 2023; What Works Clearinghouse, 2022), and assess the risk of bias accordingly.

##### Calculators

For the calculations, we will use either an Excel spreadsheet with the above formulas (using full, unrounded values for numeric calculations) or an onLine software tool called Practical Meta‐Analysis Effect Size Calculator (Wilson, 2023). The choice will depend on the type of data extracted from the included studies. In synthesizing effect sizes, we will align the effect direction across effect sizes such that the positive effect direction refers to the extent to which the intervention develops self‐regulation. Studies reporting medians and interquartile ranges may have done so for skewed data. In such cases, it is possible to conduct a meta‐analysis on a log‐transformed scale, although log‐transformed and non‐transformed data cannot be mixed in a meta‐analysis (Deeks et al., 2023).

Since we expect few studies to report medians and interquartile ranges, we will attempt to use means and standard deviations if we assume that the true distribution of outcomes in these studies is symmetric. If the above data are missing, we will either attempt to convert the data reported in the studies (e.g., statistics) to Hedges’ *g*, or contact the authors to obtain the necessary data. Although we cannot summarize the missing results of eligible studies in a meta‐analysis, we will present these studies with available information (e.g., group sample sizes) along with the primary results of the meta‐analysis. Because we are interested in the intention‐to‐treat effect (i.e., the effect of assignment), for studies that conduct intention‐to‐treat analyses, we will use the sample size at the time of enrollment in the study for estimation. However, if some participants are excluded from the analysis (i.e., available case analysis), we will use data only from participants whose outcomes are known and consider the potential impact of missing data when assessing the risk of bias.

Finally, while it is ideal to calculate summary statistics for each study in the same way (Deeks et al., 2023), this may be unrealistic due to differences in how statistics are reported in studies and the different priorities we set when extracting statistics from different study designs (i.e., summary statistics vs. adjusted effect estimates). As described above, when calculating effect estimates from the summary data, we will use the change‐from‐baseline scores to remove variability between‐individuals in the baseline. Because the standard deviations used to standardize post‐intervention scores (i.e., between‐individual variability) reflect different variability than the standard deviations used to standardize change‐from‐baseline scores (i.e., both within‐individual and between‐individual variability), we will not pool *SMD*s calculated from post‐intervention scores with those from change‐from‐ baseline scores (Deeks et al., 2023). Nevertheless, we will combine effect estimates based on change‐from‐baseline scores with effect estimates that have been statistically adjusted for baseline scores (e.g., by analysis of covariance or ANCOVA) to make full use of the available data. In this regard, we will conduct sensitivity analyses to assess the impact of using different calculations to generate effect estimates (J. P. T. Higgins, Li, et al., 2023).

#### 3.3.5 Unit of analysis issues

We are interested in intervention effects at the individual level. Thus, if we find cluster‐RCTs or individual‐assignment studies with clustering where the intervention effect was incorrectly estimated with an analysis that ignores clustering (i.e., unit‐of‐analysis error), we will attempt to re‐estimate the effect and *SE*, as described in the previous section "Measures of treatment effect". We will attempt to correct the original sample sizes with the design effect even if the values of an intraclass correlation coefficient (*ρICC*) are below 0.05 (J. P. T. Higgins, Eldridge, et al., 2023). Because we use change‐from‐baseline scores, the ideal *ρICC* is an estimate of the relative variability within and between clusters based on change‐from‐baseline scores. However, it is unrealistic to obtain *ρICC* for change‐from‐baseline scores. Alternatively, we will use *ρICC* at any time point of measurement (i.e., pre‐intervention scores, post‐intervention scores, or the scores in follow‐up evaluations) as long as the *ρICC* is based on the same target population and measurement scale as the outcome of interest. If the *ρICC* is not provided, we will ask the study authors to provide *ρICC* estimates. We will avoid adopting *ρICC* from external sources that use the same measurement scale, as *ρICC* can vary across populations. If studies ignore clustering but report effect estimates that are adjusted for baseline characteristics or missing data, we will use these effect estimates in a meta‐analysis. If re‐estimation is not possible, or the *ρICC* does not fully account for other potential sources of clustering, we will report the effect estimate and include the notation “unit‐of‐analysis error” in the risk of bias assessment. We will perform a sensitivity analysis to examine the robustness of the adjusted meta‐analysis results.

Unit of analysis issues can arise when multiple outcomes or multiple intervention groups within studies are included in a meta‐analysis (J. P. T. Higgins, Eldridge, et al., 2023). Although conventional meta‐analyses tend to use approximations of the variances of synthetic effect sizes (i.e., the averages of multiple effect sizes), we attempt to account for correlated (e.g., multiple outcomes within studies) and hierarchical (e.g., multiple intervention groups within studies) dependencies among effect sizes. Following Pustejovsky and Tipton's recommendation (Pustejovsky & Tipton, 2021), we will use a three‐step analysis procedure consisting of (1) identifying a working model, (2) estimating meta‐regression coefficients assuming the working model is true, and (3) calculating standard errors and hypothesis tests using robust variance estimation (RVE). For this purpose, we will use the packages “metafor” (Viechtbauer, 2010, 2023) and “clubSandwich” (Pustejovsky, 2023) in R. Although the metafor package's rma.mv () command does not allow for the Hartung‐Knapp method of improved estimates for hypothesis testing and confidence intervals (Hartung & Knapp, 2005), we will fit the model using a t‐distribution as an approximation (Viechtbauer, personal communication, October 30, 2021).

Multiple publications are another problem. If we find multiple reports of the same study, we will link these reports so that a meta‐analysis is based on independent findings. We will correspond with the study authors if there is any ambiguity.

#### 3.3.6 Dealing with missing data

We will contact the authors of the included studies to obtain missing outcome data for the meta‐analysis. If we are unable to obtain the data, we will take this into account when assessing the risk of bias and present the studies with missing results along with the results of the meta‐analysis (e.g., forest plots).

#### 3.3.7 Assessment of heterogeneity

We will examine the potential sources of heterogeneity between studies in the primary meta‐analysis using a meta‐regression analysis. As with the primary analysis, we will use random‐effects meta‐regression to account for residual heterogeneity between intervention effects that are not modeled by the explanatory variables.

#### 3.3.8 Assessment of reporting biases

We will assess whether studies selectively excluded outcomes that the study authors proposed to measure in either the methods section or the study protocol. In addition, we will assess the risk of reporting bias using either RoB2 (for RCTs) or ROBINS‐I (for NRSIs) and examine the impact on effect estimates in the primary meta‐analysis through sensitivity analyses.

**Figure 17:**
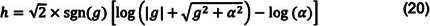
Formula for variance‐stabilizing transformation of Hedges' *g* statistic in funnel plot analysis.

If we find 10 or more eligible studies, we will use a funnel plot (i.e., plotting effect estimates against the standard error of the effect estimate) to examine potential bias due to non‐reporting, bias due to small studies, or bias due to poor methodological quality of included studies (Page et al., 2023). *SMD*s are known to be inherently correlated with their standard errors, which can lead to asymmetry in a funnel plot (Zwetsloot et al., 2017) and increase the Type I error rate in commonly used methods such as Egger's regression test (Pustejovsky & Rodgers, 2019). Therefore, we will perform a visual inspection of a funnel plot using a variance‐stabilizing transformation for a *g* statistic (*h*) using formula 20 (Pustejovsky & Rodgers, 2019; Wilson, 2017; see Figure 17).

**Figure 18:**
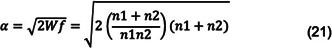
Formula for defining *α* with variance contributions and degrees of freedom.

Here, *α* is defined by formula 21 (see Figure 18), where *W* is the contribution of the variance of the approximately unbiased sample estimates of the mean difference between two groups and *f* is the degrees of freedom corresponding to the estimated standard deviation (Pustejovsky & Rodgers, 2019; Wilson, 2017).

**Figure 19:**
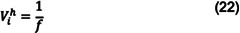
Formula for sampling variance of transformed effect size.

The sampling variance of the transformed effect size can be expressed by formula 22 (see Figure 19).

Following the R code provided by Pustejovsky and Rodgers, we will attempt to run Egger's regression test (using an additive random effects model and the Knapp‐Hartung adjustment) and the three‐parameter selection model based on the transformed effect size and sampling variance. Note that we will combine multiple effect sizes within studies so that the same funnel plot shows an effect size from the same study. Therefore, we plan to create a funnel plot for the primary outcome.

#### 3.3.9 Data synthesis

We will attempt to synthesize effect estimates from a heterogeneous group of self‐regulation interventions. To this end, we have planned random‐effects meta‐analyses using generic inverse‐variance weighted averages for primary and secondary outcomes (Deeks et al., 2023). We will use a random‐effects method that assumes that studies estimate systematically different but related intervention effects rather than the same underlying intervention effects across studies (Deeks et al., 2023). In addition, we will combine the results of RCTs and NRSIs in a meta‐analysis, assuming that both study designs address sufficiently similar research questions and have no systematic differences in the PICO elements. Overall, we plan to conduct the following two meta‐analyses:

1. A primary analysis of the intervention effects on self‐regulation
2. A secondary analysis of the intervention effects on academic skills

**Figure 20:**
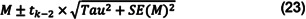
Formula for creating prediction interval as indicator of heterogeneity in random‐effects meta‐analysis.

The meta‐analyses will provide the summary estimate (i.e., the center of the distribution of intervention effects) and the confidence interval (i.e., the uncertainty in the location of the mean of the systematically different effects), although the confidence interval in a random‐effects meta‐analysis does not represent the degree of heterogeneity across studies (Deeks et al., 2023). Thus, if we can assume a normal distribution of effects between studies (i.e., no clear asymmetry of the funnel plot for more than 10 studies), we will create a prediction interval as an indicator of heterogeneity using formula 23 (Deeks et al., 2023; see Figure 20), where *M* is the summary mean of the random‐effects meta‐analysis, *t*_*k‐2*_ is a 95th percentile *t*‐statistic of a *t*‐distribution with *k*−2 degrees of freedom, *k* is the number of studies, *Tau* ^*2*^ is the estimated extent of heterogeneity, and *SE*(*M*)^2^ is the standard error of the summary mean.

**Figure 21:**
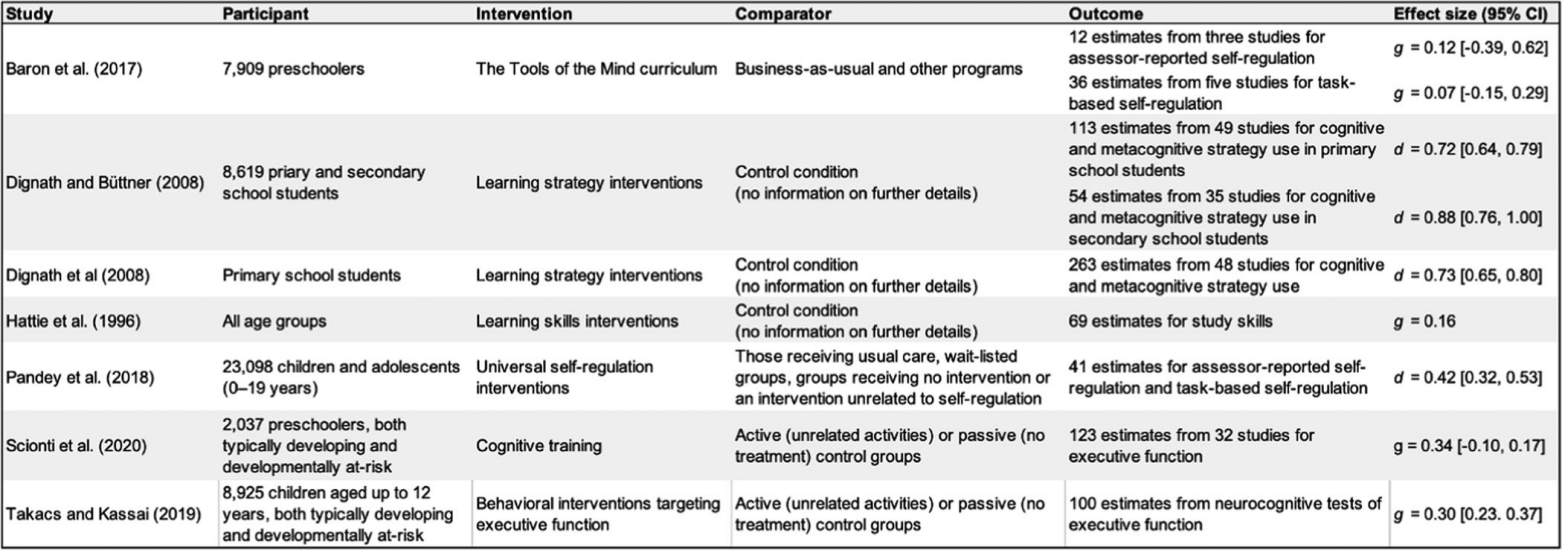
Summary effect sizes for self‐regulation reported in previous meta‐analyses.

**Figure 22:**
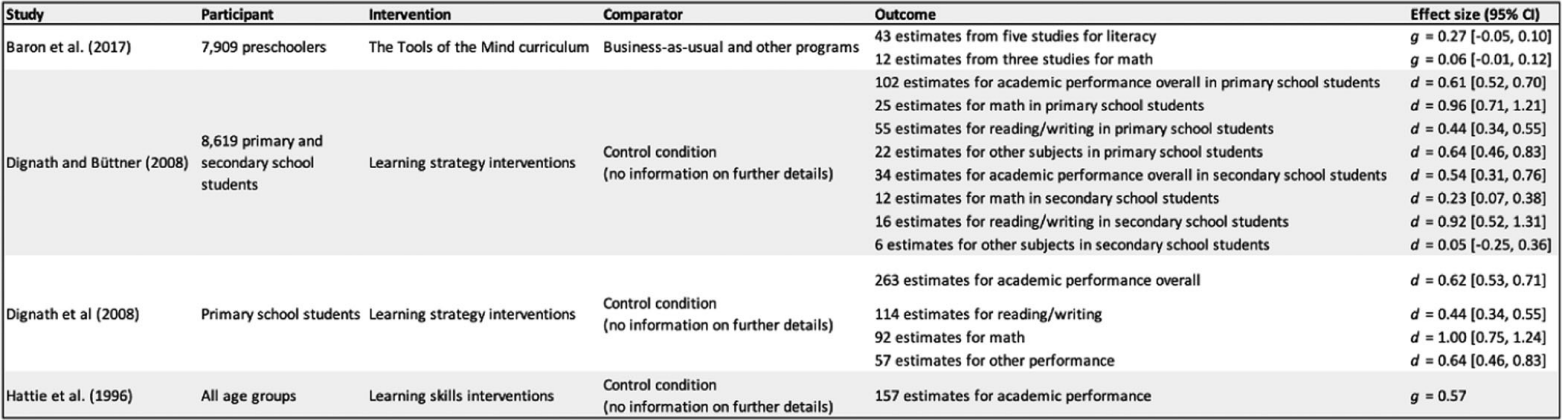
Summary effect sizes for academic skills reported in previous meta‐analyses.

Previous summary effect sizes reported in existing meta‐analyses are shown in Figure 21 (for self‐regulation) and Figure 22 (for academic skills). Despite the plan, we will forgo meta‐analyses for outcomes reported by fewer than two studies (R. Ryan, 2016) or not include studies in the meta‐analyses from which *SMD*s and standard errors were not available.

#### 3.3.10 Subgroup analysis and investigation of heterogeneity

We are interested in the following characteristics of the interventions (see the section “How the intervention might work for rationale”):

1. Resource Allocation
  a. Period
  b. Volume
  c. Duration
  d. Frequency
  e. Adherence
  f. Class Size
  g. Number of Adults
  h. Pupil‐Teacher Ratio
2. Activity Type
  a. Social Cognitive Theory‐Based Activities
  b. Motivational Theory‐Based Activities
  c. Socio‐Cultural Theory‐Based Activities
  d. Physical Activities
  e. Musical Activities
  f. Pretend Play Activities
  g. Construction Play Activities
  h. Story‐Based Activities
  i. Mindfulness‐Based Activities
  j. Academic Activities
3. Instructional Method
  a. Role of Instructor
  b. Method of Instruction
  c. Type of Feedback
  d. Fading of Instructional Support
  e. Task Complexity Adjustment
  f. Performance‐Based Rewards
  g. Engagement‐Based Rewards

If we find more than 10 eligible studies with the outcome data of interest, we will attempt to conduct a multivariate meta‐regression analysis using RVE with Resource Allocation, Activity Type, and Instructional Method as three sets of explanatory variables. After running a baseline model, we will add the first set of moderators (i.e., Resource Allocation), followed by Activity Type and Instructional Method. We will then look at the change in between‐study variance from one model to another to understand the importance of the newly added moderators on self‐regulation. This analysis will include all the aforementioned moderators to provide a more robust and nuanced model.

While our primary approach is designed for comprehensive analysis, we acknowledge the potential for alternative strategies under specific conditions. These could include insufficient degrees of freedom or an imbalance in the number of studies across different moderators. In such cases, we may opt for separate univariate meta‐regressions for each set of moderators using RVE.

Although the inclusion of each covariate is predetermined based on its assumed positive effect, we are aware that the multiplicity of analyses leads to an increased probability of false‐positive findings (Baker et al., 2009); the risk of false‐positives would be greater than 60% (i.e., 1–0.95^19^; Thompson & Higgins, 2002). Rather than relying solely on the significance level adjusted by a strict Bonferroni correction, we will focus on:

1. The variation in Hedges’ *g* across sub‐groups, expressed as a regression coefficient
2. The 95% confidence interval for the regression coefficient, specifically examining whether the interval includes zero
3. The reduction in the heterogeneity indicator, either *H* ^2^ or the ratio of overall variability of observed outcomes to sampling variability, in comparison to the intercept model
4. The inclusion of at least two studies for each category in dummy variables offers preliminary insights into the significance of each covariate

By adopting this approach, we aim to balance rigor and flexibility in our analytical strategy.

#### 3.3.11 Sensitivity analysis

We will assess the robustness of the conclusions based on key assumptions or variations in the primary and secondary meta‐analyses. To do this, we will compare the results of different versions of a meta‐analysis, perform meta‐regressions to compare sub‐groups of studies and present a summary table of the results. We will limit the sensitivity analyses to a selection of potentially influential arbitrary decisions that play a role in this review.

Specifically, we will examine the following alternative choices:

##### Analytical approach

◆ Small‐study effects

We will compare studies with relatively small samples to other studies to examine whether the average effect in random‐effects analysis is inflated by studies with small samples. The sample size threshold will be set depending on the studies included.

◆ Methods for calculating the *SMD*

Consistency in the methods used to calculate *SMD* is highly desirable. However, to combine the best available evidence on the review questions across a heterogeneous group of studies, we chose to combine Hedges’ *g* from both change‐from‐baseline scores (based on summary statistics) and effect estimates adjusted for confounding, clustering, and missing data. Thus, we will run three separate univariate meta‐regressions to compare the results with and without adjustments for confounding, clustering, and missing data. Confounding and missing data may bias the summary estimates and reduce their precision. On the contrary, because the studies that ignore clustering tend to receive more weight than they should, the results that ignore clustering may have artificially narrower confidence intervals around the effect estimates (J. P. T. Higgins, Thomas, et al., 2023).

##### Study backgrounds

◆ Study design

We will conduct a univariate meta‐regression to test the validity of the decision to combine the results of RCTs and NRSIs in a meta‐analysis. If we find systematic differences in effect estimates between RCTs and NRSIs, we will conduct a multivariate meta‐regression of the summary estimate with Study design (RCTs vs. NRSIs), Risk of bias (lower risk of bias vs. higher risk of bias), and interaction term (i.e., Study design × Risk of bias) to examine whether these differences can be attributed to differences in risk of bias between RCTs and NRSIs or to the lack of directness of NRSIs in answering the review questions.

◆ Risk of bias

We will use univariate meta‐regression to compare the results of meta‐analyses that include or exclude studies at high risk of bias. If we find that the decision to include the studies at high overall risk of bias results in an invalid summary effect estimate and a substantial increase in heterogeneity, we will reduce confidence in the conclusion of the meta‐analysis. Based on the sensitivity analysis of the effects of study design and risk of bias, we will discuss the key features of the study design that may lead to valid estimates of intervention effects in future research.

##### Outlying studies

To evaluate the influence of potential outliers on our meta‐analysis, we will conduct dedicated sensitivity analyses that include and exclude these studies. Outliers will be systematically identified using statistical diagnostics such as Cook's distance and leverage values, as well as through visual inspection of forest and funnel plots. Upon identification, we will assess their impact on key metrics such as the overall effect size and between‐study heterogeneity. If the inclusion of outliers substantially distorts these metrics, they may be excluded from the main analysis. The outcomes of these sensitivity analyses will be meticulously documented and reported to offer a nuanced understanding of the robustness of our meta‐analytic findings and provide insights into the stability of the results when subjected to the influence of outlying data points.

In addition, we plan to conduct univariate meta‐regressions to examine the consistency of results across subgroups of the following study characteristics:

##### Operationalization

◆ Self‐regulation construct (i.e., self‐regulation vs. self‐regulated learning vs. the integration of executive functions or effortful control)
◆ Self‐regulation system (i.e., cool self‐regulation vs. hot self‐regulation vs. cool and hot self‐regulation)
◆ Domain specificity (i.e., domain‐general self‐regulation vs. domain‐specific self‐regulation)
◆ Online self‐regulation measure vs. offline self‐regulation measure
◆ Direct self‐regulation measure vs. indirect self‐regulation measure
◆ Academic skills construct (i.e., emergent literacy skills vs. math skills)

##### Participant characteristics

◆ Country
◆ Age
◆ Gender
◆ Socioeconomic status
◆ Ethnicity
◆ Language learning status

In the meta‐regressions, we will not adjust the significance level because our goal is to informally assess how different methods, assumptions, definitions, and subgroups may affect the conclusions of the meta‐analyses based on the magnitude and direction of the effect estimates without relying solely on statistical significance (Page et al., 2021; Thabane et al., 2013).

#### 3.3.12 Summary of findings and assessment of the certainty of the evidence

We will assess the quality of evidence for the primary outcome using the Grading of Recommendations Assessment, Development, and Evaluation (GRADE) approach. In general, it is recommended that the initial level of confidence for NRSIs be rated as low. However, because we plan to use the ROBINS‐I instrument, which assesses the risk of bias due to lack of randomization, we will assume a high level of certainty for both RCTs and NRSIs. The GRADE approach either downgrades the quality of evidence based on the five domains of certainty (i.e., the risk of bias; consistency; directness; precision; publication bias) or upgrades it for three additional domains (i.e., the presence of large effects; gradient intervention effects; good control for confounders). The results of the GRADE assessment will be presented in a “Summary of findings” table along with the summary effect and described with quaLifiers such as “will” (high certainty), “probably” (moderate certainty), “may” (low certainty), “we are uncertain” (very low certainty) to indicate the level of certainty (Santesso et al., 2020; Schünemann et al., 2023).

AK will take the lead in utilizing the GRADEpro GDT software (GRADEpro GDT, 2024) to assess the results of all studies on an outcome‐by‐outcome basis, classify the certainty of a body of evidence for the summary effect, produce a “Summary of findings” table. In parallel, other available reviewers will independently assess the results of their designated sections within the included studies, in line with our work allocation strategy employed in earlier stages of the review. Should any ambiguities or discrepancies arise, they will be collaboratively addressed and resolved by the team.

## CONTRIBUTIONS OF AUTHORS

Atsushi Kanayama, Iram Siraj and Mariola Moeyaert are the principal investigators for this study and are responsible for overseeing and managing the study. Their combined expertise in the relevant content and methodological approaches ensures the robustness and validity of the entire review process.

Kat Steiner, our information retrieval specialist, conducts an extensive literature search. With her expertise, she ensures that the most relevant and important sources are considered.

The review team, consisting of Elie Chingyen Yu, Katharina Ereky‐Stevens, Kaoru Iwasa, Moeko Ishikawa, Mehar Kahlon, Rahel Warnatsch, Andreea Dascalu, Ruoying He, Pinal Mehta, Natasha Robinson, and Yining Shi, is responsible for the individual sections of this review. Their tasks range from preliminary research and data collection to assessing the risk of bias and determining the certainty of the evidence collected. With their extensive experience in conducting rigorous scientific research, these co‐reviewers use their broad knowledge and skills to make informed decisions in their respective areas of responsibility.

## DECLARATIONS OF INTEREST

The authors have no conflicts of interest.

## SOURCES OF SUPPORT

### Internal sources

♦ No sources of support provided

### External sources

♦ No sources of support provided

### PEER REVIEW

The peer review history for this article is available at https://www.webofscience.com/api/gateway/wos/peer-review/10.1002/cl2.1383.

## Supporting information

Supporting information.

## Data Availability

Data openly available in a public repository that issues DOIs
