# Supplementary material for: PROTOCOL: Key characteristics of effective preschool‐based interventions to promote self‐regulation: A systematic review and meta‐analysis
Source: Campbell Syst Rev. 2024 Apr 2;20(2):e1383. doi: 10.1002/cl2.1383 (PMC10985547; doi:10.1002/cl2.1383)
Supplement: Supplementary file 1 — Supporting information. [file CL2-20-e1383-s001.docx]

# Appendices

## Appendix 1. The interplay of environmental, sociocultural, and individual covariates in self-regulation development

### Introduction

The development of self-regulation in children, which is an essential prerequisite for a variety of outcomes such as academic achievement, mental health, and social skills, is a process influenced by a complex interplay of environmental, sociocultural, and individual factors. The understanding of self-regulation has expanded over time. It is based on general systems theory and was later further developed by models that emphasize both the internal control processes of the individual and the role of the social context. Influential meta-theories such as bioecological systems theory and relational and developmental systems theory have further elaborated the symbiotic relationships between the individual and the multi-layered contexts in which they exist. The aim of this review is to present a comprehensive examination of environmental, sociocultural, and individual covariates that have the potential to act as confounders in studies examining interventions aimed at promoting self-regulation in children. The focus is on aspects that have been empirically investigated within the micro and macro systems of Bronfenbrenner's ecological systems theory.

### Conceptual foundation and sociocultural perspectives in self-regulation development

The concept of self-regulation has its roots in the general systems theory of the late 1940s and early 1950s and emerged as an attempt to unravel the properties of systems - or phenomena - that remain relatively stable over time (Guttman 1991; Vancouver 2000). The cybernetic model of self-regulation represents this view, even though it focuses primarily on the internal control processes of individuals.

In contrast, sociocultural models of self-regulation accentuate the role of the social context. These models assume that self-regulation develops through social interactions with more proﬁcient individuals, such as adults and peers (Stetsenko 2009; Williford 2013). Thus, infants initially rely on their caregivers for feeding, warmth, and comfort until they eventually gain the ability to self-regulate these basic biological processes. When children enter preschool, they gradually adopt social norms, such as sharing toys, attending to teachers, and following instructions in the classroom. Sociocultural models therefore assume that the regulation of psychological and social processes by others plays a central role in the maturation of young children's self-regulation (see Figure 23; Sameroff 2009).


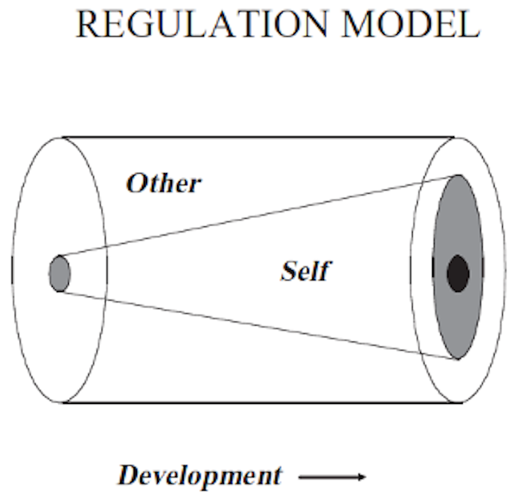


Figure 23

Change in the balance between self-regulation and other-regulation through development. Reprinted from “Conceptual Issues in Studying the Development of Self-Regulation” by Sameroff 2009, in *Biopsychosocial Regulatory Processes in the Development of Childhood Behavioral Problems* (pp. 1-18), edited by A. J. Sameroff and S. L. Olson, 2009, Cambridge University Press. Copyright 2009 by Cambridge University Press.

In a study examining the social evolution of self-regulation in community activities, Grau and Whitebread conducted an in-depth case study with eight primary school children aged eight to nine in Chile (Grau 2012). Their discourse analysis illustrated how shared regulation helped a group of children formulate goals, track their progress, and evaluate their academic task performance. However, the study did not provide compelling evidence of how shared regulation promoted the development of individual self-regulation in children.

### Applying a pragmatic approach to understanding self-regulation in bioecological and relational-developmental contexts

Two influential meta-theories, Bioecological Systems Theory (Bronfenbrenner 1979; Bronfenbrenner 1986) and Relational-Developmental Systems Theory (Overton 2013), offer comprehensive insights into the development of self-regulation, focusing particularly on the intricate interplay between individual characteristics and the surrounding social environment.

Bioecological Systems Theory frames human development within ﬁve environmental strata: micro-, meso-, exo-, macro-, and chronosystems, each reflecting different levels of interaction within the individual's environment (see Figure 24; for a detailed explanation of each level see Bronfenbrenner 2005). Relational-Developmental Systems Theory, on the other hand, views development as the result of symbiotic interactions between individuals and the multi-layered contexts in which they exist (McClelland 2015; Overton 2013).


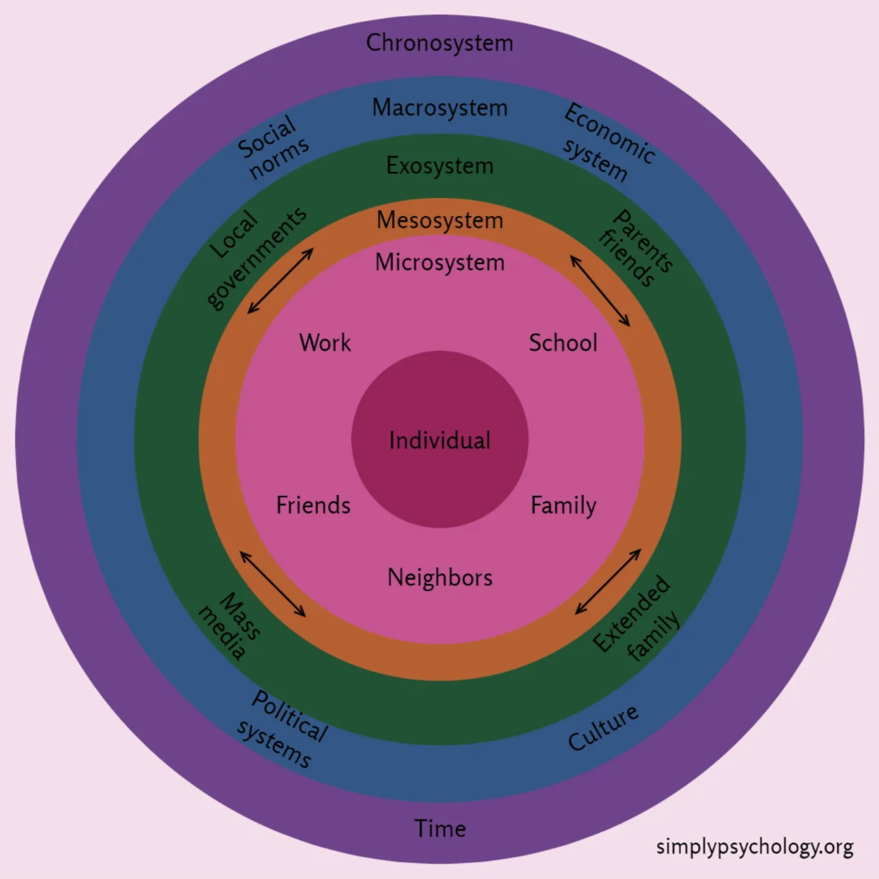


Figure 24

The five ecological systems. Adapted from "Bronfenbrenner’s ecological systems theory" by Guy-Evans (2024, January 17), SimplyPsychology. Retrieved from <https://www.simplypsychology.org/bronfenbrenner.html>.

These two meta-theories differ primarily in their ontological perspectives. While Overton's theory presupposes a directionality in human development, such as a speciﬁc order or sequence, Bronfenbrenner's theory does not adhere to this principle (Tudge 2016). Notwithstanding this divergence, both theories reject the reductionist notion of isolating cause-effect relationships from other contributing variables, a stance seemingly at odds with the aims of this review.

However, the application of a pragmatic approach can provide a philosophical framework that can reconcile these theoretical discrepancies (Johnson 2004; Tashakkori 1998). According to Johnson and Onwuegbuzie, a pragmatic perspective values knowledge that is practically applicable and therefore suggests that researchers should integrate methods and philosophical viewpoints that are most appropriate for answering the research questions posed (Johnson 2004). Similarly, Tashakkori and Teddlie value the flexibility that pragmatism offers researchers in exploring their research interests (Tashakkori 1998).

For the purposes of this review, we will therefore selectively refer to the micro and macro systems of Bronfenbrenner's theory, focusing in particular on aspects whose relevance to the development of child self-regulation has been empirically investigated.

### From micro to macro: exploring influences on child self-regulation across Brofenbrennerʼs ecological systems

Bronfenbrenner's ecological systems theory suggests a reciprocal relationship between children and their immediate environment, microsystem, including elements such as family, school, neighborhood, and other care settings. The macrosystem, a broader environmental layer, encompasses elements such as the child's ethnicity, the parent's socioeconomic status, and cultural values, which interact with the child's characteristics and influence routine actions within the microsystem (Vélez-Agosto 2017). This perspective aligns with the prevailing research literature.

A comprehensive meta-analysis examining the role of parenting practices on child behavior, which utilized 41 studies, identiﬁed a positive correlation between positive control (deﬁned as directive parental behavior complemented by encouragement and clear guidance) and child compliance (Karreman 2006). Conversely, negative control was inversely associated with preschool children's compliance, indicating that harsh or overly restrictive practices may impede a child's willingness or ability to comply (Karreman 2006).

Interestingly, this study did not reveal a statistically significant relationship between parental responsiveness and child compliance or between the three aspects of parenting (positive control, negative control, and responsiveness) and inhibitory control or emotion regulation (Karreman 2006). These ﬁndings suggest that the impact of parental responsiveness on child compliance and the influence of these parenting aspects on children's impulse control or emotional regulation may not be as substantial as anticipated.

Another meta-analysis revealed a significant positive relationship between effortful control, a facet of self-regulation, and a secure attachment (Pallini 2018). This suggests that children with a greater capacity for effortful control are more likely to form secure attachments. Prior research has also demonstrated a relationship between secure attachment and parental responsiveness (Behrens 2011), suggesting that responsive parents are more likely to foster secure attachment in their children, potentially enhancing self-regulation development.

In line with the importance of positive parenting practices, Meuwissen and Carlson conducted an experimental study that highlighted the role of parent autonomy support in enhancing preschoolers’ self-regulation. The study found that even brief interventions could signiﬁcantly alter parental behavior, particularly in parents who initially exhibited lower levels of autonomy support. These changes were subsequently reflected in the children’s self-regulation abilities (Meuwissen 2019). This suggests that parent autonomy support could be a crucial covariate to control for when examining the effects of preschool-based interventions targeting self-regulation.

Supporting this, there is increasing empirical evidence of a positive association between elements of positive parenting (including the home learning environment, parental involvement, parental scaffolding, autonomy support, parental control, warmth, and belief) and the development of executive functions and self-regulation in children (Fay-Stammbach 2014; Sanders 2019). These relationships persist after controlling for potential confounders such as age, gender, and IQ (Díaz 2017; Meuwissen 2019; Roskam 2014; Wong 2008), suggesting a signiﬁcant and direct influence of positive parenting practices on children's cognitive development and self-regulation abilities.

Extensive empirical studies have investigated the complex relationships between self-regulation in children and diverse childrearing environments, encompassing factors such as household chaos, media exposure, socioeconomic conditions, and cultural differences (Bridgett 2015; McClelland 2018). Prior research indicates that environmental instability, such as household chaos, can adversely affect a child's self-regulatory abilities (Bronfenbrenner 2000). Crespo and colleagues further elucidated this relationship, showing that self-regulation acts as a buffer against the negative behavioral outcomes associated with household chaos (Crespo 2019). This ﬁnding complements a meta-analysis by Andrews and others, which revealed a signiﬁcant inverse relationship between household chaos and the growth of effortful control and executive function (Andrews 2021). These insights collectively suggest that interventions aimed at enhancing self-regulation could be especially beneﬁcial for children in chaotic households.

As our society becomes increasingly digital, the importance of fostering self-regulation in children's use of technology and media literacy has been underscored (Campbell 1999; Leaton Gray 2022). Research indicates that children with emerging self-regulation can become engrossed in television and electronic games (Cliff 2018), exacerbating self-regulation problems, especially in early formative years. The interplay between increased media exposure and its effects on self-regulation is particularly pronounced among children from socioeconomically disadvantaged backgrounds (Radesky 2014; Ribner 2017).

The impact of socioeconomic status on the development of self-regulation also appears to be connected to a child's language acquisition status. For example, Wanless and associates found that in the United States, English-speaking children from lower socioeconomic backgrounds eventually caught up with their peers from higher socioeconomic backgrounds in self-regulation (Wanless 2011). However, this pattern was not observed among children for whom English is a second language, suggesting socioeconomic conditions' effects on self-regulation development may vary with language learning status.

Culture further complicates the relationships between self-regulation and factors such as child characteristics, parental education, and environmental influences. Differences in self-regulation between genders have been observed in several countries, with girls generally exhibiting better abilities than boys (Matthews 2009; Matthews 2014, p. 201). However, in France and some Asian countries, no signiﬁcant gender differences were found in self-regulation on direct measures, but teachers still rated girls' self-regulation more favorably than boys'. Cultural values and potential caregiver biases may influence these perceptions (Acar 2019; Broekhuizen 2015; Matthews 2014; Wanless 2013).

### Conclusion

Drawing from the complex web of factors explored in this review, it has become apparent that promoting children's self-regulation necessitates a multi-faceted approach. Interventions should be designed considering the intricate interplay of child factors such as gender, ethnicity, and language learning status, parental factors such as socio-economic status and parenting practices, and environmental influences such as household chaos, media exposure, and culture.

Our analysis underscores the urgency of implementing targeted strategies, including comprehensive education programs, parent workshops, school-based interventions, and related policy initiatives. These strategies must be particularly attentive to at-risk groups, advocate for balanced media consumption, and prioritize the fostering of child self-regulation.

As our study speciﬁcally addresses universal preschool-based interventions aimed at self-regulation, the above factors serve as significant covariates that could potentially confound the effects of such interventions. The accurate estimation of intervention effects, therefore, hinges on careful control and consideration of these covariates. This underscores the essential need for interventions to be tailored and sensitive to the unique needs and backgrounds of each child, especially since evidence suggests that children with the lowest baseline levels of self-regulation stand to gain significantly from such personalized interventions (Bierman 2008; Diamond 2011; Diamond 2012).

In conclusion, this review serves as a vital call to action for researchers, educators, and policymakers to recognize and leverage the potential of targeted, early interventions that are specifically crafted to promote children's self-regulation. By acknowledging the multi-faceted nature of self-regulation and the array of factors influencing its development, we are better positioned to prepare our children with the necessary competencies to successfully navigate life’s challenges. Doing so promises to foster a future generation characterized not only by resilience and adaptability but also by a profound sense of autonomy and well-being.

## Appendix 2. Search strategies

For detailed search strategies used in this review, refer to: [Search strategy for each electronic database](https://docs.google.com/spreadsheets/d/1r9Tifm8-eVG7kIRnYI5rBOIzVN6mpYow_pFIjqhqUdU/edit?%20usp=sharing)

## Appendix 3. Coding instructions

For the coding instructions applied in the analysis, refer to: [Coding instructions](https://docs.google.com/document/d/19HkQ62zkUitCZMhHp_PJjKTf8_F9xh8k3c2UFDY-MEg/edit?usp=sharing)
